# Ferutinin induces osteoblast differentiation of DPSCs via induction of KLF2 and autophagy/mitophagy

**DOI:** 10.1038/s41419-022-04903-9

**Published:** 2022-05-12

**Authors:** Jyotirindra Maity, Derek Barthels, Jaganmay Sarkar, Prateeksha Prateeksha, Moonmoon Deb, Daniela Rolph, Hiranmoy Das

**Affiliations:** Department of Pharmaceutical Sciences, Jerry H. Hodge School of Pharmacy, Texas Tech University Health Sciences Center, Amarillo, TX USA

**Keywords:** Macroautophagy, Mitophagy

## Abstract

Osteoblast differentiation is critically reduced in various bone-related pathogenesis, including arthritis and osteoporosis. For future development of effective regenerative therapeutics, herein, we reveal the involved molecular mechanisms of a phytoestrogen, ferutinin-induced initiation of osteoblast differentiation from dental pulp-derived stem cell (DPSC). We demonstrate the significantly increased expression level of a transcription factor, Kruppel-like factor 2 (KLF2) along with autophagy-related molecules in DPSCs after induction with ferutinin. The loss-of-function and the gain-of-function approaches of KLF2 confirmed that the ferutinin-induced KLF2 modulated autophagic and OB differentiation-related molecules. Further, knockdown of the autophagic molecule (ATG7 or BECN1) from DPSC resulted not only in a decreased level of KLF2 but also in the reduced levels of OB differentiation-related molecules. Moreover, mitochondrial membrane potential-related molecules were increased and induction of mitophagy was observed in DPSCs after the addition of ferutinin. The reduction of mitochondrial as well as total ROS generations; and induction of intracellular Ca^2+^ production were also observed in ferutinin-treated DPSCs. To test the mitochondrial respiration in DPSCs, we found that the cells treated with ferutinin showed a reduced extracellular acidification rate (ECAR) than that of their vehicle-treated counterparts. Furthermore, mechanistically, chromatin immunoprecipitation (ChIP) analysis revealed that the addition of ferutinin in DPSCs not only induced the level of KLF2, but also induced the transcriptionally active epigenetic marks (H3K27Ac and H3K4me3) on the promoter region of the autophagic molecule ATG7. These results provide strong evidence that ferutinin stimulates OB differentiation via induction of KLF2-mediated autophagy/mitophagy.

## Introduction

Bone is a dynamic tissue, that constantly undergoes remodeling due to the resorptive efforts of osteoclasts and bone-building effects of osteoblasts [1]. In healthy skeletal tissue, osteoclasts secrete acids and proteolytic enzymes to break down the matrix of old bone, which is then replaced by osteoblasts, which secrete the mineral and organic materials to rebuild bone [2]. Osteoblasts are the principal cells that configure bone and are derived from mesenchymal precursor cells. Osteoblastic differentiation is a lifelong process that is critically important for the development, maintenance, and recovery of bone. Various disease states compromise bone quality and can lead to severe and debilitating conditions, from arthritis to osteoporosis.

Dental pulp stem cells (DPSCs) are mesenchymal precursor cells that reside within the pulp of the third molar teeth. They possess a mesenchymal phenotype that is preserved in vitro and can be differentiated along the osteogenic, adipogenic, and chondrogenic lineages [3]. Differentiation from DPSCs is regulated in part by estrogen signaling. Estrogen plays a very crucial role in bone homeostasis by directing osteocytes, osteoclasts, and osteoblasts, which leads to inhibition of bone remodeling or diminishing bone resorption and stimulating the bone generation, respectively [4]. Ferutinin is a naturally occurring non-steroidal phytoestrogen. It shows strong agonist property for nuclear estrogen receptor alpha (ERα) whereas both agonist and antagonist properties for nuclear estrogen receptor beta (ERβ) [5]. It was documented that ferutinin brings epigenetic alterations in DPSCs and thus changes cellular pathways that stimulate osteoblastic differentiation [6]. Ferutinin also shows antioxidant, anti-inflammatory, antiproliferative, and cytotoxic activity [7], and exhibits membrane depolarization, permeability transition pore formation, and respiration uncoupling in cells [8].

Autophagy mainly macroautophagy (herein, referred to as autophagy) is an evolutionary conserved intracellular catabolic process used by cells to degrade damaged proteins or organelles and repurpose degraded biomolecules among them maintaining cellular homeostasis. It is an important mediator of various cellular functions like maintaining energy balance [9, 10] and physiological processes including aging [11, 12]. Failure of autophagy is responsible for the onset of different age-associated diseases including various neurodegenerative disorders [13] and cancer [14]. Recent studies suggest autophagy plays a crucial role in maintaining bone homeostasis through osteoclastic differentiation [15] as well as osteoblastic differentiation [16]. Moreover, it is also recorded that autophagy is induced in osteoblasts during bone mineralization, and autophagy-deficient osteoblasts secrete higher levels of receptor activator of NFκB ligand (RANKL), a critical promoter of osteoclastic differentiation and function [17].

Mitochondria are very dynamic organelles and are known as the cells’ powerhouse for ATP production [18]. Its function, quality, and quantity, are necessary for the maintenance of cellular survival. It fuses and divides throughout its lifespan depending upon the requirement of the cell [19]. Cells try to maintain a healthy mitochondrial network utilizing several quality control pathways. However, if mitochondria are damaged is beyond repair, mitochondria are engulfed by autophagosomes and degraded via fusion with lysosomes known as mitophagy [20, 21]. Mitophagy has an enormous role in cellular maintenance and differentiation from progenitor cells [16]. In terms of dealing with osteoporosis or other bone degenerative diseases, the finding of new drugs or natural products is essential to induce osteoblast differentiation from its progenitor cell. Recent research revealed that Vitamin K2 favors MC3T3‑E1 cells towards osteoblast differentiation by mineralization via autophagy induction [22]. Earlier, the role of kaempferol in the differentiation and mineralization of osteoblastic MC3T3-E1 cells via autophagy was also reported [23].

Kruppel-like factor 2 (KLF2) is a zinc-finger transcription factor. First discovered in the lung, it is essential for blood vessel development during embryogenesis [24]. While it is best known for its atheroprotective roles, it has recently been shown to promote cell development and to play an important role in the cellular response to inflammatory stimuli [25–27]. A growing body of work reveals that KLF2 plays a profoundly important role in bone biology [28–31]. Recent findings from our lab show that KLF2 plays an important role in regulating autophagy during osteoclastic and osteoblastic differentiation from different progenitor cells suggesting that the molecule has a significant impact on autophagy-mediated cellular differentiation [15].

Here, we provide the first evidence of a phytoestrogen, ferutinin-mediated induction of KLF2, and autophagy that favors the osteoblastic differentiation of DPSCs. We have established the involvement of both KLF2 and autophagy by using gain-of-function and loss-of-function approaches for both the KLF2 gene and two essential autophagic genes such as ATG7 and BECN1. These findings shed light on ferutinin as a pharmacological compound to target KLF2 in modulating autophagy, which could be used to develop a new potential regenerative therapeutic approach to bone disorders, such as arthritis and osteoporosis.

## Results

### Effect of ferutinin on osteoblast differentiation-related proteins in DPSCs

**Fig. 1:**
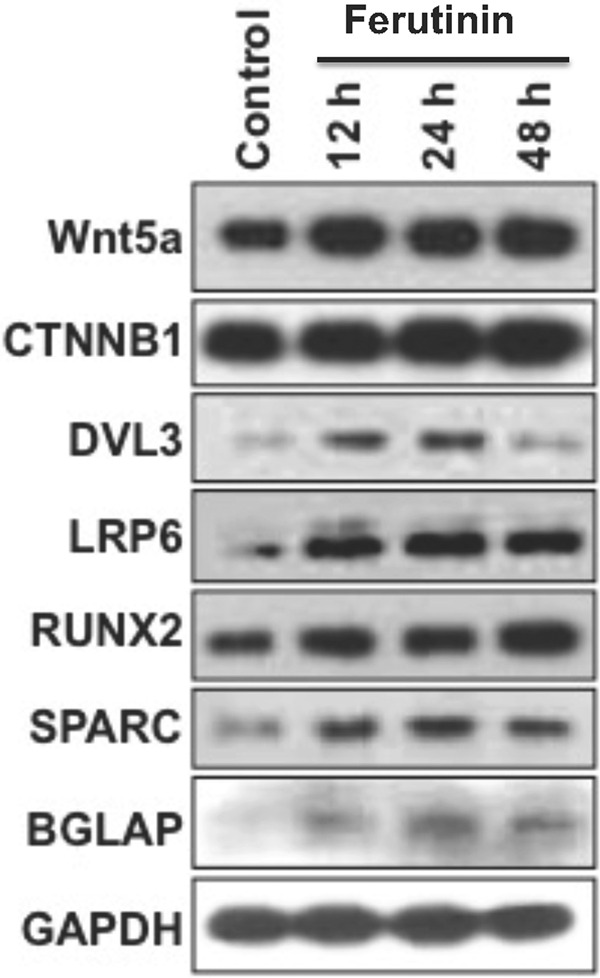
Ferutinin induces osteoblast differentiation-related molecules in DPSCs. Ferutinin was added to the DPSCs and cells were harvested at various time points as stated, whereas, vehicle-treated DPSCs were considered as a control. Western blot images are shown for various osteoblast marker proteins, and the GAPDH was considered as an internal loading control (*n* = 3).

Treatment with ferutinin-induced expression of Wnt/β-catenin pathway proteins associated with osteoblastogenesis (Fig. 1). Specifically, increased expression levels of Wnt5a, β-catenin, and LRP6 were observed at 12, 24, and 48 h of stimulation. Dvl3 expression levels were elevated at 12 and 24 h of treatment. Ferutinin also increased the expression of Runx2, a key transcription factor associated with the BMP2 (bone morphogenetic protein 2) pathway, in a time-dependent manner. Expression levels of the functional proteins osteonectin and osteocalcin were also increased following treatment with ferutinin. These findings indicate that phytoestrogen ferutinin promotes osteoblastic induction in DPSCs.

### Effect of ferutinin on autophagic vesicles formation, expression of autophagic proteins, and KLF2 in DPSCs

**Fig. 2:**
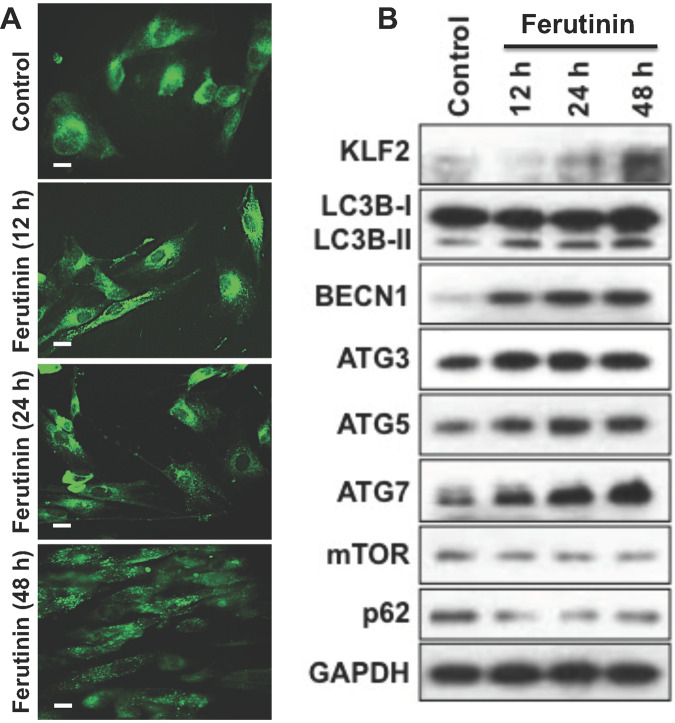
Ferutinin induces KLF2, autophagic vesicles, and autophagy-related molecules in DPSCs. **A** Ferutinin was added to the DPSCs and cells were subjected to the monodansylcadaverine (MDC) staining at various time points. Autophagic vesicles were more prominent in the later time points (*n* = 3). **B** Western blot images of the KLF2 and autophagy-related proteins were shown at various time points overexpression, and the GAPDH was considered as an internal loading control (*n* = 3).

MDC staining of ferutinin-treated DPSCs revealed that the number of autophagic vesicles in treated cells increases in a time-dependent manner (Fig. 2A). Protein expressions of autophagy-related molecules were increased over time in ferutinin-stimulated DPSCs (Fig. 2B). Specifically, LC3B-II expression was increased overall at three treatment time points, as did Beclin1, ATG5, and ATG7. A slight increase in ATG3 expression level was observed, though the change was not substantial. Inhibition of mTOR is an earmark of autophagy induction [32]. We also observed decreased protein expression of mTOR and p62 after ferutinin treatment to DPSCs; indicating the induction of autophagy. Taken together, these results reveal ferutinin-induced autophagy in DPSCs.

### Effect of knockdown of Klf2, Atg7, and Becn1 on autophagy and osteoblastic markers in DPSCs cultured with ferutinin

**Fig. 3:**
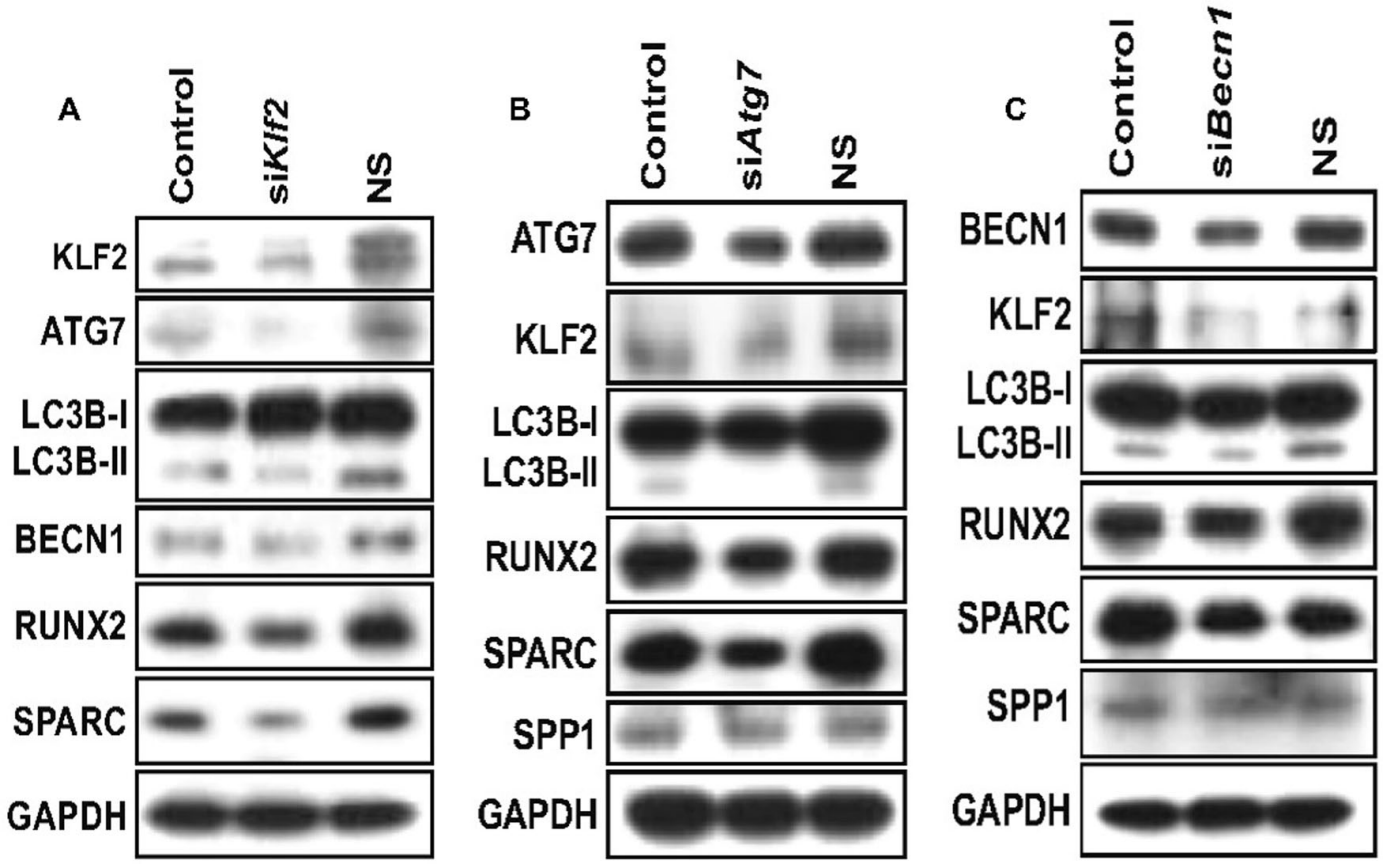
Knockdown of KLF2 or ATG7 or BECN1 reduced autophagy-related molecules and osteoblast differentiation-related molecules in DPSCs. KLF2 or ATG7 or BECN1 was knocked down in DPSCs when cells were cultured in the ferutinin-containing medium. **A** Western blot images for detection of autophagy-related molecules after knockdown of KLF2. **B** Western blot images for detection of autophagy-related molecules and osteoblast differentiation-related molecules after knockdown of ATG7. **C** Western blot images for detection of autophagy-related molecules and osteoblast differentiation-related molecules after knockdown of BECN1. GAPDH was considered as an internal loading control in every experiment (*n* = 3).

Next, we became curious to determine the consequences of KLF2 knockdown during DPSCs cultured with ferutinin. More than 70% knockdown was achieved after the siRNA-mediated knockdown of KLF2. However, partial knockdown of KLF2 resulted in significantly decreased levels of autophagic proteins (ATG3, ATG5, ATG7, LC3B-II: LC3BI ratio, BECN1) (Fig. 3A). These findings prove the positive correlation between KLF2 with autophagic molecules as well as OB differentiation-related molecules (Fig. 3A). We next put effort to determine whether autophagy-related molecules also have a reciprocal effect on KLF2 or not, by knocking down crucial autophagy-related molecules ATG7 and BECN1 in DPSCs. Knockdown of ATG7 resulted in reduced autophagy as evidenced by reduced levels of LC3B-II: LC3BI ratio (Fig. 3B). Notably, partial knockdown of ATG7 resulted in downregulation of KLF2 protein level as well as OB differentiation-related marker proteins like RUNX2, SPARC (osteonectin), and SPP1 (osteopontin) (Fig. 3B). Similarly, knockdown of BECN1 inhibited autophagy-related molecules, specifically the ratio of LC3B-II: LC3BI. This knockdown resulted in a reduced level of KLF2 also, and concomitantly reduced levels of OB differentiation-related marker proteins including RUNX2, SPARC, and SPP1 (Fig. 3C). Together, these results show the positive correlation between KLF2 and autophagy-related molecules along with OB differentiation-related molecules.

### Effect of ferutinin on mitochondrial membrane potential in DPSCs

**Fig. 4:**
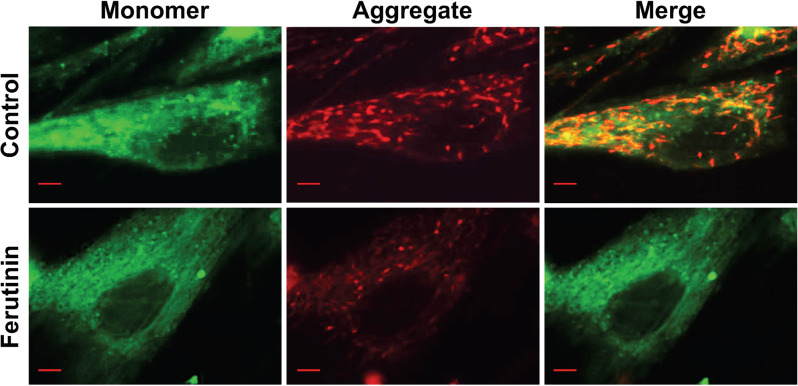
Ferutinin reduces mitochondrial membrane potential in DPSCs. Ferutinin was added to the DPSCs and after 24 h cells were subjected to JC1 staining to detect mitochondrial membrane potentials. Images were captured for single cell. Controls were the vehicle-treated DPSCs. Scale bar = 10 μM (*n* = 3).

The mitochondrial membrane generates an electrochemical proton gradient to drive ATP synthesis [33]. The interior of the mitochondria is electronegative, which drives the influx of cations and efflux of anions. Mitochondrial membrane potential was evaluated in ferutinin-stimulated DPSCs via staining with fluorescence conjugated JC1 dye. JC1 is a lipophilic cationic dye, which naturally produces green fluorescence. When it is internalized into mitochondria, it forms reversible J aggregate complexes that produce red fluorescence. Hence, in cells with healthy mitochondrial membrane potential, red-stained spots are observed, whereas cells undergoing decreased membrane potential, exhibit largely green fluorescence. In our study, untreated DPSCs stained with JC1 dye exhibited stained spots of red throughout the cytoplasm (Fig. 4). Following 24 h of treatment with ferutinin, less red-stained spots were observed as compared to untreated control. This result indicates a possible reduction of mitochondrial membrane potential in ferutinin-treated DPSCs.

### Effect of ferutinin on production of ROS, and intracellular Ca^2+^ in DPSCs

**Fig. 5:**
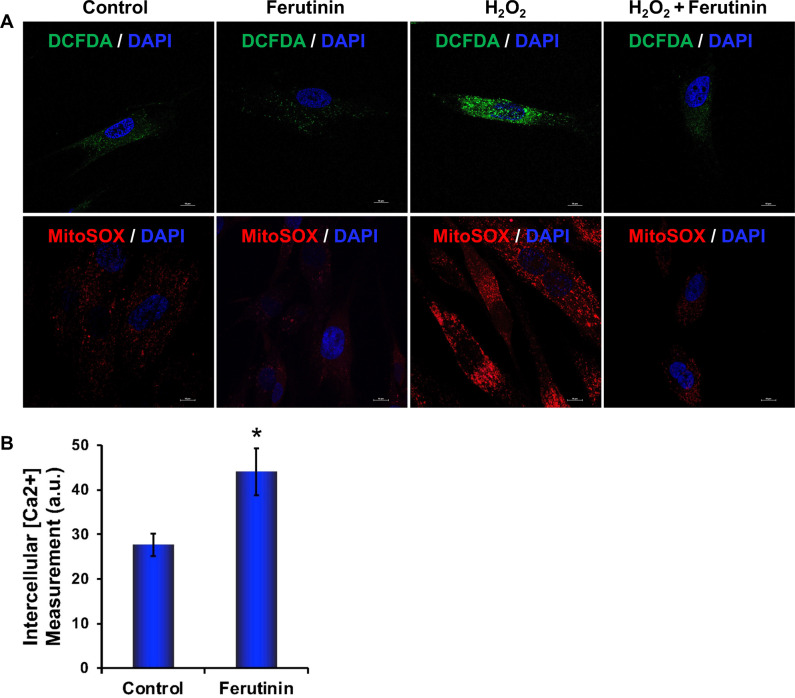
Ferutinin reduces ROS in DPSCs both in basal and activated conditions, and induces intracellular Ca^2+^. **A** ROS generation was detected by DCFDA and mitoSOX staining both in basal and activated condition (in presence of H_2_O_2_). **B** The intracellular Ca^2+^ production was measured in DPSCs after addition of ferutinin to the cells for 24 h. Vehicle-treated DPSCs were considered as control. (*) indicates a statistical significance (*p* < 0.05) when compared with ferutinin-treated cells to the control cells (*n* = 3).

To investigate the status of ROS generation during the OB differentiation after stimulation with ferutinin in the presence or absence of H_2_O_2_ (an inducer of ROS) using DCFDA and mitoSOX staining. We observed that the level of DCFDA and mitoSOX staining was remarkably decreased in DPSCs after the addition of ferutinin in both basal as well as during the H_2_O_2_ treated conditions (Fig. 5A). This result reveals that ferutinin can reduce oxidative stress in DPSC.

Calcium plays an important role in bone remodeling. Both extracellular and intracellular Ca^2+^ play crucial roles that are involved in the regulation of cell proliferation and differentiation [34]. Elevated extracellular Ca^2+^ concentrations modulate MSCs behavior towards bone formation [35]. In osteoblasts, the elevation of intercellular Ca^2+^ is due to a higher release of Ca^2+^ from the endoplasmic reticulum or also simultaneously influx of Ca^2+^ via voltage-gated Ca^2+^ channels from extracellular matrix [36]. We also observed that the level of intracellular Ca^2+^ was significantly increased in DPSCs after addition of ferutinin for 24 h (Fig. 5B).

### Effect of ferutinin on mitochondrial structures and on the expression of mitophagic molecules

**Fig. 6:**
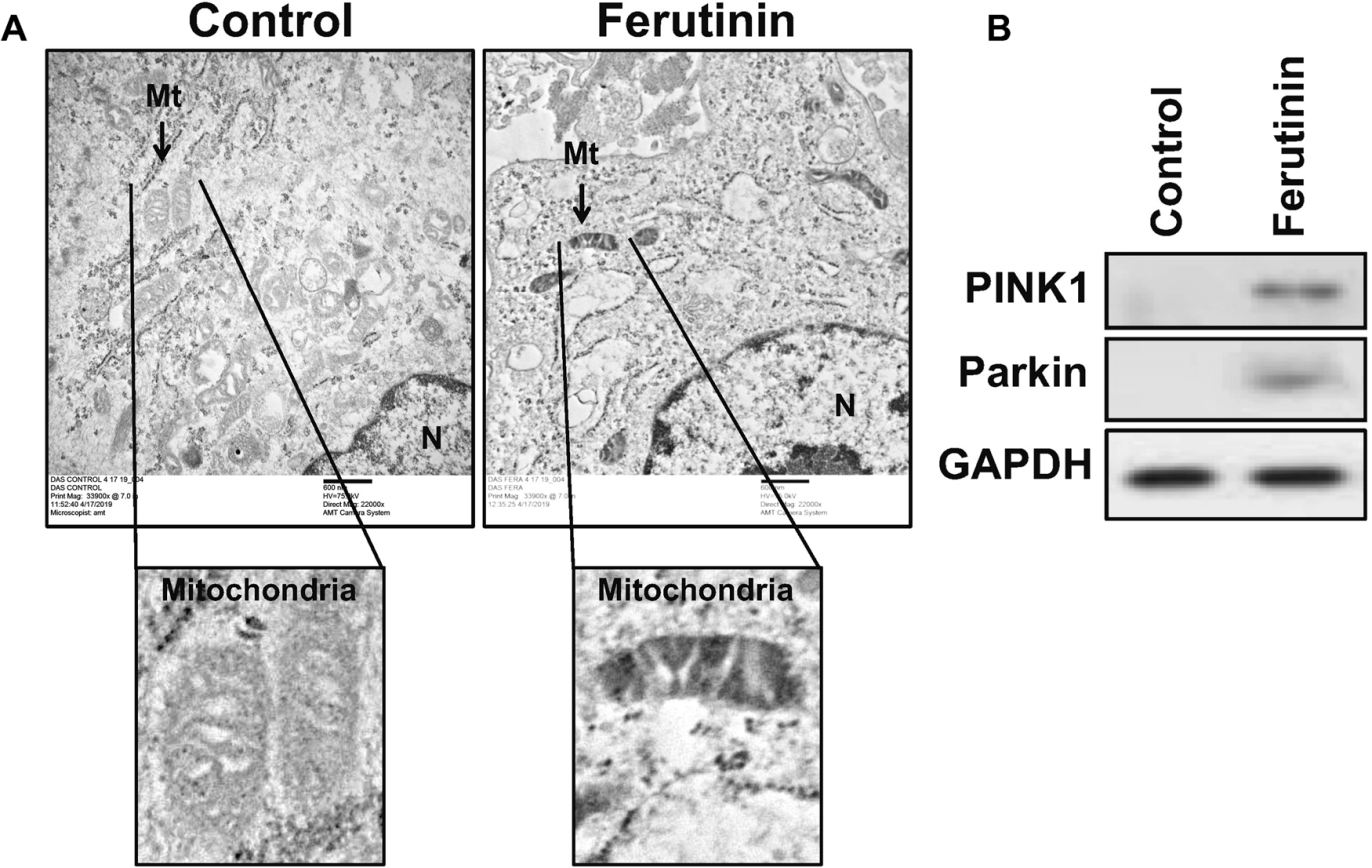
Ferutinin distorts healthy mitochondrial structures towards mitophagy in DPSCs. **A** Ferutinin was added to the DPSCs, and after 24 h cells were subjected to transmission electron microscopic (BioTEM) analysis to detect mitochondrial ultrastructural morphology. Areas of specific interest were blown to show mitochondrial ultrastructure. **B** Similarly, ferutinin was added to the DPSCs. After 24 h cells were harvested and collected total proteins were subjected to western blot analysis for mitophagy marker molecules, and the GAPDH was considered as an internal control (*n* = 3).

Mitophagy is the process where damaged mitochondria are engulfed within autophagosomes and catabolized by fusion with lysosomes [37]. Bio-transmission electron microscopy (Bio-TEM) revealed that untreated DPSCs possess healthy mitochondria (Fig. 6A). However, following 24 h of treatment with ferutinin, mostly distorted and damaged mitochondria were observed. Moreover, in Fig. 6A engulfed mitochondria within the vacuole were also visible. These images indicate the involvement of induction of mitophagy in ferutinin-stimulated DPSCs. On the other hand, Parkin acts as a downstream effector molecule of PINK1 and favors mitophagy [20]. We observed no expression of PINK1 or Parkin in untreated DPSCs (Fig. 6B). Whereas, after 24 h ferutinin treatment, expression of both PINK1 and Parkin proteins were increased (Fig. 6B). This finding further suggests that ferutinin promotes mitophagy in DPSCs and favors OB differentiation.

### Effect on extracellular acidification rate in DPSCs after addition of ferutinin

**Fig. 7:**
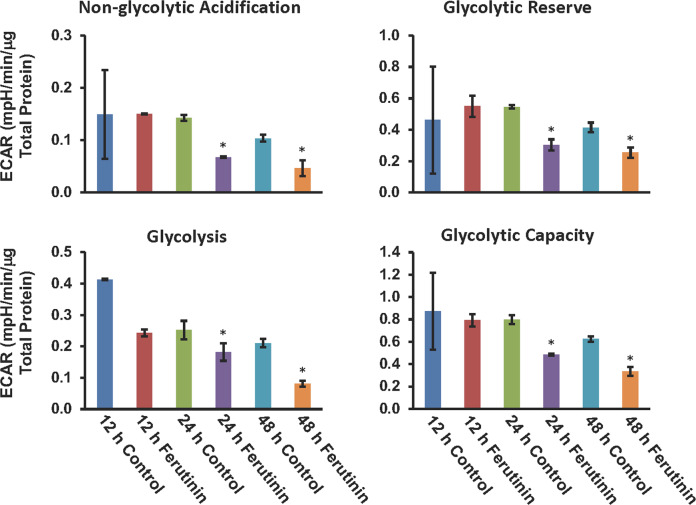
Reduced extracellular acidification rate in DPSCs after addition of ferutinin. Seahorse extracellular flux analysis was performed to evaluate the extracellular acidification rate (ECAR) in the non-glycolytic acidification, glycolytic reserve, glycolysis, and the glycolytic capacity conditions in DPSCs after the addition of ferutinin at various time points. Vehicle-treated DPSCs were considered as a control for a particular time point (*n* = 3).

We next became interested in investigating the effects of ferutinin on the process of mitochondrial respiration, so we performed “glycolysis stress tests” on DPSCs after the addition of ferutinin to the DPSCs. We initially tested at 24 h time point. Later we extended the experiment to include 12 h and 48 h time points to ensure our findings are consistent. The cells treated with ferutinin showed lower extracellular acidification rates (ECAR) than that of their vehicle-treated counterparts (Fig. 7). This trend was observed with all parameters examined, including non-glycolytic acidification, glycolysis, glycolytic capacity, and glycolytic reserve. We found a significant decrease in the level of non-glycolytic acidification at both 24 and 48 h of ferutinin treatment. In addition, glycolytic reserve, glycolysis, and glycolytic capacity were also significantly decreased after the addition of ferutinin to the DPSCs at both 24 and 48 h time points (Fig. 7). During differentiation, an increase in glycolytic activity was expected [16]. However, after the addition of ferutinin, which initiates osteoblastic differentiation of DPSCs, a decrease in glycolytic activity was found. These results are in line with our other observations where we found that the mitochondria were damaged after the addition of ferutinin to the cells. This could be a potential explanation for the reduced level of glycolytic activity observed in DPSCs after the addition of ferutinin.

### Effect of ferutinin in the epigenetic activation marks on Atg7 promoter regions

**Fig. 8:**
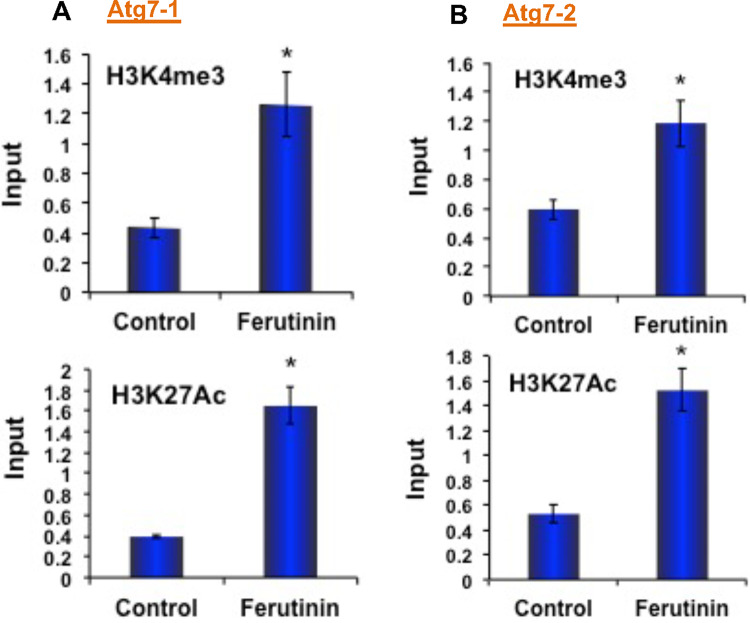
Ferutinin regulates the ATG7 gene epigenetically in DPSCs. Autophagy signaling pathway molecule ATG7 was analyzed using KLF2-ChIP and quantitative PCR methods after the addition of ferutinin to the DPSCs for 24 h. Active marks of the ATG7 gene for both H3K4me3 and H3K27Ac molecules were evaluated in the promoter site using two different primers such as **A** ATG7-1 and **B** ATG7-2, and shown graphically. Vehicle-treated DPSCs were considered as a control. Star (*) indicates a statistical significance (*p* < 0.05) when compared ferutinin-treated cells to the controls (*n* = 3).

Trimethylation of histone 3, lysine 4 (H3K4me3), and acetylation of histone 3 lysine 27 (H3K27Ac) are signs of active transcription [38, 39]. After the demonstration of *Atg7* expression is directly correlated with *Klf2* expression, we became curious to investigate the epigenetic regulatory mechanisms of the *Atg7* gene during OB differentiation. From the human UCSC genome browser, we found that the enrichment sites for epigenetic marks are situated around +0.5 kb (upstream) of the *Atg7* transcriptional start site (TSS). We examined in-depth, to find out whether any epigenetic involvement of *Klf2* binding to the promoter region of *Atg7* and the regulation of its’ expression. we performed a *Klf2*-chromatin immunoprecipitation (ChIP) analysis in ferutinin-stimulated DPSCs along with keeping unstimulated DPSCs as a control (vehicle-treated control). We used two different sets of primer sequences from two different regions of the *Atg7* promoter (Fig. 8A, B). In both sets, in control cells, *Klf2* binding was detected at the promoter region of *Atg7*. Whereas, *Klf2* binding was notably increased in ferutinin-stimulated DPSCs. In addition, the levels of H3K4me3 and H3K27Ac marks were also significantly increased in ferutinin-stimulated DPSCs (Fig. 8A, B). Taken together these data confirm that *Klf2* expression was not only increased in ferutinin-stimulated DPSCs but also binding efficiency of *Klf2* was increased in *Atg7* promoter region, resulting in activation of *Atg7* gene through the enrichment of H3K4 methylation as well as H3K27 acetylation. Thus, these findings indicate that ferutinin brings essential epigenetic changes and favors osteoblastic differentiation of DPSCs.

## Discussion

Osteoblast differentiation has enormous importance in the regulation of bone homeostasis, and dysregulation leads to bone and cartilage pathologies. Many efforts were given to find out the mechanisms of OB differentiation from precursor MSCs, though the mechanism is still elusive. On a different note, autophagy is well known for its critical role in retaining mesenchymal stemness as well as cellular differentiation [40]. As a cytoplasmic phenomenon, it recycles metabolic precursors and aids in cellular energy balance during the basal and stressed conditions. It was shown that autophagy and KLF2 share common activation and regulatory pathways to maintain endothelial phenotype and survival [41]. In addition, induction of KLF2 along with autophagy in prostate cancer cells was also documented [42]. Furthermore, a conserved KLF-autophagy pathway was noted in nematode lifespan and mammalian age-associated vascular dysfunction [43]. Moreover, studies from our lab showed that KLF2 can negatively or positively regulate autophagy during osteoclast and osteoblast differentiation respectively [15]. We have undertaken a current study to dissect the various aspects of molecular mechanisms ferutinin (phytoestrogen)-mediated initiation of OB differentiation in DPSCs. This research was aimed to provide a new insight for the development of improved therapeutics and modulate treatment options for bone degenerative diseases.

We have shown recently that the induction of autophagy benefits osteoblast cell differentiation from DPSC using the classical osteoblastic differentiation method [16]. Herein, after treatment with ferutinin, we also noticed that upregulated autophagic markers, LC3B-II, BECN1, ATG3, ATG5, and ATG7 along with downregulation mTOR and p62 in DPSCs, which indicates the induction of autophagy (Fig. 2). It is interesting to observe that the induction of KLF2 appears to occur later than the induction of the autophagy-related markers. Several possibilities might explain this phenomenon. It is possible that the KLF2 expression at the very low level might have a much bigger effect on autophagic protein expressions. There is also a possibility of involvement of other molecules along with KLF2 during ferutinin-mediated initiation of osteoblast differentiation of DPSC. All these phenomena are under investigation, and yet to be conclusively defined. However, maximum induction of autophagy proteins (ATG7, LC3B, BECN1) were after 48 h of treatment of ferutinin, which are concomitant with the maximum KLF2 protein expression level. To find out the effect of KLF2 on ferutinin-mediated initiation of OB differentiation, we performed loss-of-function experiments with KLF2 and revealed a positive correlation as observed reduction of autophagy (LC3B-II, BECN1, ATG3, ATG5, and ATG7) markers (Fig. 3A). In consistent with our findings, recent evidence suggests that the upregulation of KLF2 during OB differentiation from mouse pre-osteoblastic cells due to the KLF2-mediated enhanced transcriptional activity of RUNX2 [44]. Then we put effort to investigate the effect of autophagy on KLF2 expression. We targeted two different autophagy-related molecules, such as ATG7 and BECN1 for the siRNA-mediated inhibition of autophagy. It is noteworthy, that inhibition of autophagy via both molecules reduced the KLF2 expression (Fig. 3B, C). Another important observation to be noted here is that the BECN1 knockdown also showed a reduction in the level of KLF2 expression along with inhibition of autophagy. This phenomenon might be due to the feedback loop mechanism during KLF2-driven autophagy during initiation of osteoblast differentiation of DPSC as well as starvation and rapamycin-mediated autophagy induction [16]. This finding indicated a confirmed role of autophagy-mediated KLF2 regulation during ferutinin-stimulated initiation of OB differentiation of DPSCs.

WNT5A-mediated osteoblast differentiation in MSCs [45] and bone formations are well-established [46]. We investigated the effect of ferutinin on Wnt signaling molecules during the induction of OB differentiation in DPSCs. We observed the increased levels of WNT5A, CTNNB1 (β-Catenin), DVL3, and LRP6 after 12 h ferutinin treatment. This data re-confirms the involvement of the Wnt pathway during ferutinin-stimulated induction of OB differentiation, similar to the classical pathway of OB differentiation using ascorbic acid and β-glycerophosphate [16]. These findings reconfirm our previous data showing ferutinin directs OB differentiation of DPSCs through the Wnt signaling pathway [6], and involvement of Wnt signaling-induced Warburg effect, which is an activator of OB differentiation [47]. Studies from our lab and others showed that the Wnt-mediated osteoblast differentiation has occurred via inhibition of GSK3 protein. Our current understanding is that the effect of ferutinin on Wnt signaling may vary depending on cell types. In this study, we found that the involvement of transcription factor KLF2, however, participation of other molecules cannot be ruled out, thus it can be considered either KLF2 dependent or KLF2 independent, which is yet to be determined. Earlier publication from our lab showed that the induction of autophagy via treatment with rapamycin (mTOR inhibitor) or starvation resulted in upregulation of KLF2 [16] asserted the positive correlation between autophagy and KLF2 in DPSCs. It was shown that estrogen could promote autophagy in osteoblasts during differentiation via upregulation of RAB3GAP1 [48]. Thus, these further highlight possible crosstalk among autophagy, KLF2, and Wnt signaling during ferutinin-mediated initiation of osteoblast differentiation.

Calcium (Ca^2+^) plays a crucial role in bone formation. The osteoblast plays a central role in regulating skeletal structure along with maintaining calcium homeostasis [49]. We found an elevated intracellular calcium level after ferutinin stimulation in DPSCs (Fig. 5B), which is aligned with the earlier findings indicating the increased intercellular calcium during OB differentiation [50]. Recent data identified that mitochondrial damage occurs through elevated production of ROS, mitochondrial superoxide, and protein oxidation, which can inhibit OB differentiation [51]. We found a lesser amount of ROS and mitochondrial superoxide production in ferutinin-treated DPSCs, which demonstrated that DPSCs maintain healthy mitochondrial status by diminishing ROS levels. We were further curious to find how the ROS level was reduced and its’ possible defense mechanism during the initiation of OB differentiation. It is known that mitophagy plays a pivotal role in clearing damaged mitochondria, and control ROS production in growing cells under stress conditions, thus ensure cell survival. Cellular ROS level has an impact on the initiation of autophagy [52]. We found a lower level of mitochondrial membrane potential (MMP) after ferutinin-stimulated DPSCs (Fig. 4). Decreased MMP (reduced aggregated red form) is an indicator of lower ATP production that hinders cellular differentiation. To accomplish increased demand of energy during differentiation, along with ATP production, mitochondria also generate higher ROS as an obvious effect of energy production, which damages itself [51]. Mitochondrial fission can lead to mitophagy, which is detected with the elevated level of PINK1 and Parkin expressions [53]. Observations of the increased levels of PINK1 and Parkin accumulations in ferutinin-stimulated DPSCs (Fig. 6B) confirm the occurrence of mitophagy. Ferutinin decreases the generation of total ROS production within the cell including mitochondrial ROS production both in basal and activated conditions (Fig. 5A), which is concomitant with the initiation of essential molecular signaling pathways, and finally, intracellular structural changes favor osteoblastic differentiation of DPSCs. Induction of mitophagy eliminates damaged and unnecessary mitochondria from the cell thus keeps only the healthy mitochondria, which counts a lower number of total mitochondria in the cell after addition of ferutinin. Our present understanding is that, this lower number of mitochondria favors reduced ROS generation of per healthy mitochondria thus counts overall decreased level of ROS production during initiation of OB differentiation of DPSC. Further reconfirmation, by mitochondrial structure through BioTEM was carried out, which confirmed the presence of mitophagy during ferutinin-treated DPSCs (Fig. 6A). Taking together, our findings confirmed that the ferutinin brings about metabolic changes associated with mitophagy that increased cell defense mechanism during initiation of OB differentiation of DPSCs. In addition, degradation of cytosolic damaged proteins or organelles via autophagy provides cells amino acids which are oxidized to support ATP production was already documented [53].

Previous studies published in the literature assert that the glycolytic pathway is largely responsible for the energy production that drives osteoblastic differentiation [54]. With this knowledge, we expected to see an increase in ECAR as the differentiation process progressed over time [16]. Our seahorse data trends in the opposite direction, with ECAR decreasing with the ferutinin treatments over time (Fig. 7). While unexpected, this phenomenon is not unprecedented, as previously published studies have shown similar results at the initiation of differentiation [55]. In addition, we have seen a significant reduction of mitochondrial membrane potentials and increased amount of mitochondrial damage after addition of ferutinin to the DPSCs. These mitochondrial damages might contribute to the significant reduction in glycolytic activities. This sector demands more investigations to find out the actual behavior of DPSCs during OB differentiation.

Recently, epigenetic mechanisms, such as DNA methylation or histone modifications in regulating autophagy have received great interest. KLF2 is a zinc-finger transcription factor involved in the early differentiation of cells [56]. To detect the KLF2-mediated direct regulation of autophagic pathway in ferutinin-treated DPSCs, we examined the KLF2 binding profiles around TSS of the ATG7 promoter. H3-trimethyl-Lys4 (H3K4me3) and H3-acetyl-Lys27 (H3K27Ac), are two established histone marks associated with active promoters and enhancers that initiate elongation by chromatin accessible to RNA polymerase II [57]. We have designed KLF2 primers to analyze the binding along with H3K4me3 and H3K27Ac around the TSS region of *ATG7* in ferutinin-treated DPSCs. ChIP analysis revealed that ferutinin-stimulated DPSCs were associated with the increased level of KLF2 binding along with H3K4me3 and H3K27 expressions on around TSS region of *ATG7* ferutinin-treated DPSCs (Fig. 8). Thus, taking together, ferutinin exerts its effects on DPSCs via epigenetic regulation. It can upregulate the canonical Wnt pathway in DPSCs through upregulating epigenetic modulation [6, 58]. In final mechanistic approach we found that ferutinin also influence KLF2 activation marks on the promoter of *Atg7*, indicating that it has a direct effect on the expression of KLF2 level and autophagy-related molecules during initiation of OB differentiation.

## Conclusion

In sum, herein, we provide the first evidence for the induction of KLF2 after addition of ferutinin to the DPSCs, which is corroborated with autophagy-related molecules and osteoblast differentiation-related molecules. We confirmed the involvement of KLF2 and autophagy using loss-of-function approaches for both KLF2 gene and two autophagic genes, such as *ATG7* and *BECN1*. In addition, we showed that ferutinin induced the mitophagy, and reduced the metabolic functions of mitochondria in DPSCs. We further confirmed with epigenetic studies showing that addition of ferutinin in DPSCs not only induced the level of KLF2, also induced the transcriptionally active epigenetic marks (H3K27Ac and H3K4me3) on the promoter region of the autophagic molecule ATG7. These results provide strong evidence that ferutinin stimulates OB differentiation via induction of KLF2-mediated autophagy/mitophagy.

## Materials and methods

### Reagents and antibodies

alpha (α) Modified Essential Medium (MEM, M8042-500 ML), Trypsin (25200-056), Ferutinin (SML1609-5MG), Monodansylcadaverine (MDC, D4008), JC1 dye (T3168), DCFDA (D6883), fluoro 4 (F14201), Ponceau (P7170), TEMED (161–800), and Imprint Chromatin Immunoprecipitation kit (17–295) were purchased from Sigma-Aldrich Corporation. Seahorse XF cell mito-stress kit (103035-100), Glycolysis stress kit (103020-100), XF Base Medium (102353-100), Glucose Solution (103577- 100), Pyruvate Solution (103578-100), Glutamine Solution (103579- 100) and XF calibrant (100840-000) were obtained from Agilent. Bradford reagent (500-0006) was from Bio-Rad. Fixative for TEM (15960-01) was purchased from Electron microscopy sciences. 4% Paraformaldehyde (sc-281692) was from Santa Cruz. Hanks’ balanced salt solution (HBSS, 21-020-CV) was from Mediatech Inc. si*ATG7* (AM16708), si*BECN1* (4457298), scramble si*RNA* (AM4611), si*KLF2* (4392420) and DEPC-Treated Water (AM9922) were picked up from Ambion. Alizarin Red Solution (2003999) was obtained from Chemicon international. Opti-MEM (31985), FBS (10438-026), PBS (70013-032), Pen strep (10378-016), Anti-Anti (15240), l-Glutamine (25030), Mitosox red (M36008), TRIzol reagent (15596026), cDNA kit (4387406), Lipofectamine 2000 (11668019) and mounting medium (P10144) were acquired from Invitrogen Corporation. SYBR Green PCR Kit (4309155) was from Applied Biosystem. Protogel (EC-890) was from National diagnostics. BSA (BP1600-100), 20% SDS (BP1311-1), DMSO (BP231-100), NaOH (1310-73-2497-19-8), Methanol (A412P-4) were secured from Fisher scientific. Separating buffer (BP-90), Stacking buffer (BP-95), Running buffer (BP- 150), Transfer buffer (BP-190), and TBS-T (IBB-180) were procured from Boston Bioproducts. Non-fat dry milk (M0841) was obtained from LabScientific. Antibodies for LC3B (2775), ATG3 (3415 S), ATG5 (12994 S), ATG7 (8558 S), BECN1 (3738 S), mTOR (2972 S), Wnt5a (2392), β-catenin (9562 S), DVL3 (3218 T), LRP6 (3395 T), RUNX2 (8486 S), osteonectin (8725 S) and GAPDH (2118 S), PINK1 (6946 S), Anti-mouse IgG, HRP-linked Antibody (7076), Anti-rabbit IgG, HRP-linked Antibody (7074) were obtained from Cell Signaling Technology and used as a 1:1000 dilution for western blot. RIPA lysis buffer (20–188) and osteocalcin antibody (AB10911) were purchased from Millipore. Parkin (ab77924), Osterix (ab94744), osteopontin (ab8448), H3K4me3 (ab1012), H3K27Ac (ab4729), KLF2 (ab203591), and goat IgG (ab37373) were purchased from Abcam used as a 1:1000 or 1:2000 dilution for western blot.

### DPSC isolation and expansion

Human dental pulp-derived stem cells (DPSC) were isolated from third molar teeth obtained after routine surgery from a healthy adolescent donor with advance approval from the Institutional Review Board (IRB) and consent from the donor. Teeth were washed thoroughly with phosphate-buffered saline (PBS) containing 1% Penicillin-Streptomycin-Glutamine (PSG) (Gibco, Thermo Fisher, Waltham, MA). Teeth were cut open to harvest the pulp, which was then minced into ~1 mm cubes and plated onto 60 mm cell culture dishes, in which it was cultured with alpha (α) Modified Eagle Medium (MEM) (Gibco) supplemented with 20% FBS (Hyclone, Thermo Fisher, USA) and 1% PSG. Every third day, the medium was removed, and a fresh medium was added. As cells migrated from the pulp tissue and became confluent, they were dissociated by scraping and were re-cultured as passage 1 and maintained using the same medium. Cells were maintained at 37 °C, 5% CO_2_, and 95% relative humidity. Cell viability was determined using the trypan blue exclusion method. Experiments were performed using cells between 3–9 passages.

### Gene knockdown

*KLF2* knockdown was performed with *KLF2* sequence-specific siRNA, keeping non-specific siRNA as control, and transfected with Lipofectamine 2000 (Thermo Fisher Scientific, 11668019) using 60 nmol/L concentrations as described earlier [25]. Similarly, Beclin1 and ATG5 siRNA were used for knockdown of the autophagic molecules keeping non-specific siRNA as control (Thermo Fisher Scientific), and transfected with Lipofectamine 2000 (Thermo Fisher Scientific, 11668019) using 60–100 nmol/L concentrations.

### Western blot

Whole-cell lysates were obtained from DPSCs cultured under control conditions or stimulated with ferutinin (10 μg/ml in αMEM) for 12, 24, and 48 h. Protein was quantified by colorimetric assay using the Bradford method (Bio-Rad, Hercules, CA). A polyacrylamide gel was cast and denatured proteins (20 µg each) were loaded and separated through the gel by electrophoresis and a protein ladder was loaded as a marker (Sigma, St. Louis, MO). The proteins were transferred from the gel to a 0.45 µm nitrocellulose membrane (Bio-Rad) at 4 °C. The membrane was blocked for 1 h at room temperature (RT) with a blocking buffer composed of 5% non-fat milk in TBS-Tween-20 (TBST) (Boston BioProducts, Ashland, MA). The membrane was washed and incubated with primary antibody against LRP6, Wnt5a, Dvl3, β-catenin, Runx2, GAPDH (Cell Signaling, Danvers, MA), osteocalcin, and osteonectin (Santa Cruz Biotechnology, Dallas, TX) (1:1000 diluted in a solution of 5% BSA in TBST) for 2 h. The membrane was washed, then incubated in secondary antibody (1:3000 in a solution of 5% milk in TBST) (Cell Signaling). The membrane was washed, placed in the cassette holder, and incubated briefly in the chemiluminescent substrate (Sigma). Films were then exposed and developed. Densitometric quantification of bands was performed using ImageJ software (NIH).

### MDC Staining

To determine whether autophagic vesicles were present in cells treated with ferutinin, DPSCs were grown on sterile coverslips inserted into a 6-well plate and stimulated with ferutinin or cultured under control conditions. During the course of differentiation, at 12, 24, and 48 h, cells were stained with auto-fluorescent MDC dye using a standard protocol [59]. In brief, cells were incubated with 50 mM of MDC at 37 °C for 15 min and washed three times with 1× PBS. Finally, the cells were mounted on a glass slide, viewed under a fluorescence microscope (Olympus Corporation of the Americas, Waltham, MA, Slide book 5.0 × 64 software ix81), and images were captured digitally.

### JC1 staining

DPSC cells (2 × 10^4^) were seeded in a 35 mm plate and grown overnight. The next day, cells were treated with ferutinin for 24 h. The cells were then washed 3 times with 1× PBS and incubated with JC1 dye for 20 min at 37 °C. After washing with 1× PBS, the cells were mounted on glass slides and viewed under a fluorescence microscope (Olympus Corporation of the Americas, Waltham, MA, Slide book 5.0 × 64 software ix81).

### Transmission electron microscopy (TEM)

DPSCs were treated with ferutinin for 48 h in DMEM complete medium. Cells were then harvested and prefixed with 2.5% glutaraldehyde. Further, these cells were post-fixed with 1% osmium tetra-oxide for 1 h in dark. Cells were then dehydrated by ascending concentrations of acetone. Cells were then embedded with epoxy resin. Polymerization of cells was performed by placing them gradually in an oven at 42 °C for 2 h, at 52 °C overnight, and finally at 62 °C for another overnight. Ultrathin sections (50–70 nm) of these blocks were made using a Leica Ultramicrotome EM UC6. These sections were collected from a 10% ethanol turf. The sections were contrasted using 1% aqueous uranyl acetate for 5 min and lead to citrate in a CO_2_-depleted atmosphere for 2–4 min. Hitachi H-8100 (75–200 kV) electron microscope (Japan) was deployed to evaluate the sections in 100 kV, and images were captured digitally using an AMT V700 side mount camera.

### Detection of ROS

Reactive oxygen species (ROS) detection was performed by using the fluorogenic dye 2′,7′- dichlorodihydrofluorescein diacetate (DCFDA) that enters the cells and interacts with a reactive oxygen molecule to form a green fluorescent compound dichlorodihydrofluorescein (DCF). In short, a stock solution of DCFDA (10 mM) was prepared in methanol and was further diluted with culture medium to a working solution of 100 µM. DPSCs (2 × 10^4^) were seeded in a coverslip inserted on a well of a six-well plate for overnight. The next day, cells were treated with H_2_O_2_ (200 µM, final concentration) in the presence or absence of ferutinin for 24 h. Control DPSCs were cultured for 24 h in culture medium (α-MEM) along with 10% FBS. After treatments, coverslips were washed with ice-cold Hank’s balanced salt solution (HBSS) and incubated with 100 µM of DCFDA for 30 min at 37 °C. After washing with 1 x PBS, the coverslips were mounted on glass slides. Imaging was performed under a multiphoton confocal microscope (A1R; Nikon, NY, USA), using a ×100 objective and images were analyzed using Nikon image analysis software NIS Element. Each experiment was performed in triplicate, and experiments were performed at least 3 times.

### Detection of mitochondrial ROS

Cellular mitochondrial ROS generation was evaluated by using the mitoSOX red compound (M36008, Thermo Fisher Scientific, USA). Superoxide compounds present in mitochondria, which oxidize mitoSOX red to produce red fluorescence. In short, we prepared a 5 mM stock solution of the mitoSOX in DMSO. DPSCs (2 × 10^4^) were seeded in a coverslip inserted on a well of a six-well plate overnight. The next day, cells were treated with H_2_O_2_ (200 µM, final concentration) in presence or absence of ferutinin for 24 h. Coverslips were then washed with ice-cold 1× PBS and incubated in 2 µM working solution of the mitoSOX red for 30 min at 37 °C. After washing with 1× PBS, the coverslips were mounted on glass slides. Imaging was performed under a multiphoton confocal microscope (A1R; Nikon, NY, USA), using a ×100 objective, and images were analyzed using Nikon image analysis software NIS Element. Each experiment was performed in triplicate, and experiments were performed at least three times.

### Intercellular calcium (Ca^2+^) measurement

Intracellular Ca^2+^ was measured using a fluor 4 compound (A20173, Thermo Fisher Scientific). A 1 mM fluor 4 stock solution was prepared in 1× PBS. DPSCs (2 × 10^4^) were seeded in a 10 cm dish for overnight culture. After 16 h, cells were treated with ferutinin for 24 h or left untreated. Cells were washed with ice-cold 1× HBSS three times and incubated with a 3 µM working solution of fluor 4 for 30 min at 37 °C. Cells were then washed three times with HBSS. Finally, cells were lysed with a 0.1 M NaOH alkaline solution and were harvested by scraping. 200 μl of lysate were used in each well of a 96-well plate, and fluorescence intensity reading was taken using a plate reader (Synergy 2, BioTek Instruments, Inc, Winooski, USA) at an excitation setting of 488 nm and emission setting of 520 nm. Each experiment was performed in triplicate at least three times for each assay.

### Determination of glycolysis and mitochondrial respiration

To gain a better understanding of mitochondrial function after the addition of ferutinin to DPSC, a “cell glycolysis stress” test was performed using an Agilent XFe24 machine at various time points. The final stimulating and inhibiting reagent concentrations in each well were as follows: 10 mM of glucose, 1 μM of oligomycin, and 50 mM of 2-deoxy-glucose (Glycolysis Stress Test Kit, Agilent, USA). DPSCs were seeded at a density of 2 × 10^5^ cells per well of a 24-well plate. In preparation for the assay, DPSCs were treated with ferutinin for 12, 24, and 48 h. At the 48-h time point, the DPSCs were trypsinized, centrifuged at 1400 rpm, and resuspended in normal DPSC media. Cells were counted and plated in triplicate in the Seahorse cell culture plate and allowed to adhere for 24 h before the assay was performed. On the day of the assay, assay media was prepared using Seahorse XF DMEM (Agilent, USA), supplemented with 1 mM glutamine (Agilent, USA). DPSC culture medium was removed, and each well was rinsed with assay media three times. 500 μl of assay media was then added to each well of the Seahorse cell culture plate. Cells were then incubated in a non-CO_2_ incubator at 37 °C for 1 hour before running the assay. The assay was run using a standard, unmodified ‘Glycolysis Stress’ test protocol provided by Agilent.

### Chromatin Immunoprecipitation

ChIP analysis was performed using Imprint® Chromatin Immunoprecipitation Kit (Sigma) according to previously described procedures [6]. DPSCs were either stimulated with ferutinin or grown in control conditions for 24 h. Chromatin was cross-linked with 1% formaldehyde, and DNA was sheared by sonication, then chromatin-protein complexes were immunoprecipitated with antibodies against H3K4me3 (Millipore Sigma) and H3K27Ac (Millipore Sigma). Anti-goat IgG (Abcam, Cambridge, UK) was kept as a negative control. Quantitative PCR analysis was performed with the two different primers described (in Supplementary Table-I and Fig. 1) by using SYBR green PCR master mix (Thermo Fisher) in a real-time PCR machine (Bio-Rad CFX96 Real-Time System). ChIP assay values were normalized by the background precipitation obtained with a non-specific antibody. Percent (%) of input was analyzed by use of a standard formula. Each experiment was performed at least three times.

### Statistical analysis

All experiments were performed at least three times in a triplicate manner, and the results were displayed as mean ± SEM. Statistical analyses were performed using Graph Pad Prism 5.0 for Windows (Graph Pad Software, San Diego, CA, USA). Student’s *t* test was used to perform statistical analysis of RT-qPCR and western blot graph results and *p* values less than 0.05 were considered significant (*).

## Supplementary information

Supplementary Figure Legends

aj-checklist- H Das-CDDIS-21-4692R.pdf

Original WB Scan data

Supplemental Figures

## Data Availability

The data sets used and/or analyzed during the current study are available from the corresponding author on reasonable request.
