# Supplementary material for: Ferutinin induces osteoblast differentiation of DPSCs via induction of KLF2 and autophagy/mitophagy
Source: Cell Death Dis. 2022 May 12;13(5):452. doi: 10.1038/s41419-022-04903-9 (PMC9098908; doi:10.1038/s41419-022-04903-9)
Supplement: Supplementary file 1 — Supplementary Figure Legends [file 41419_2022_4903_MOESM1_ESM.docx]

**Ferutinin induces osteoblast differentiation of DPSCs via induction of KLF2 and autophagy/mitophagy**

**Jyotirindra Maity, Derek Barthels, Jaganmay Sarkar, Prateeksha Prateeksha, Moonmoon Deb, Daniela Rolph, and Hiranmoy Das**

Department of Pharmaceutical Sciences, Jerry H. Hodge School of Pharmacy, Texas Tech University Health Sciences Center, Amarillo, Texas, USA.

**Supplementary Figures**

**Figure S1.** Bar graphs show the quantified level of Wnt5a, CNNB1, DVL3, LRP6, RUNX2, SPARC, and BGLAP expression in respect of GAPDH expression shown in Figure 1. Star (*) indicates a statistical significance (*p*<0.05) when compared stimulated DPSCs for 12, 24 or 48 h with ferutinin to controls.

**Figure S2.** Bar graphs show the quantified level of KLF2, LC3BII/LC3BI ratio, BECN1, ATG3, ATG5, ATG7, mTOR, and P62 expressions in respect of GAPDH expression shown in Figure 2B. Star (*) indicates a statistical significance (*p*<0.05) when compared stimulated DPSCs for 12, 24 or 48 h with ferutinin to controls.

**Figure S3.** Bar graphs show the quantified level of KLF2, ATG7, LC3BII/LC3BI ratio, BECN1, RUNX2, and SPARC expressions in respect of GAPDH expression shown in Figure 3A. Star (*) indicates a statistical significance (*p*<0.05) when compared KLF2 knockdown with nonspecific controls.

**Figure S4.** Bar graphs show the quantified level of ATG7, KLF2, LC3BII/LC3BI ratio, RUNX2, SPARC, and SPP expressions in respect of GAPDH expression shown in Figure 3B. Star (*) indicates a statistical significance (*p*<0.05) when compared ATG7 knockdown with nonspecific controls.

**Figure S5.** Bar graphs show the quantified level of BECN1, KLF2, LC3BII/LC3BI ratio, RUNX2, SPARC, and SPP expressions in respect of GAPDH expression shown in Figure 3C. Star (*) indicates a statistical significance (*p*<0.05) when compared *BECN1* knockdown with nonspecific controls.

**Figure S6.** Bar graphs show the quantified level of Parkin and PINK1 expressions in respect of GAPDH expression shown in Figure 5B. Star (*) indicates a statistical significance (*p*<0.05) when compared expression levels in DPSCs without or with ferutinin stimulation.

**Figure S7.** Mouse UCSC genome browser analysis for ATG7. UCSC genome browser track showing the site determination for showing selection of quantitative PCR primers for ChIP analysis. The red star (*) indicates the starting locations of quantitative PCR primer for each set.

**Figure S8.** Showing original Seahorse extracellular flux analysis graphs to evaluate extracellular acidification rate (ECAR) in the non-glycolytic acidification, glycolytic reserve, glycolysis and the glycolytic capacity conditions in DPSCs after addition of ferutinin at various time points. Vehicle-treated DPSCs were considered as a control for a particular time point (n=3).

**Table S1.** Showing primer sequences and genomic locations used in chromatin immunoprecipitation quantitative PCR assays.
