# Supplementary material for: Ferutinin induces osteoblast differentiation of DPSCs via induction of KLF2 and autophagy/mitophagy
Source: Cell Death Dis. 2022 May 12;13(5):452. doi: 10.1038/s41419-022-04903-9 (PMC9098908; doi:10.1038/s41419-022-04903-9)
Supplement: Supplementary file 3 — Original WB Scan data [file 41419_2022_4903_MOESM3_ESM.pdf]

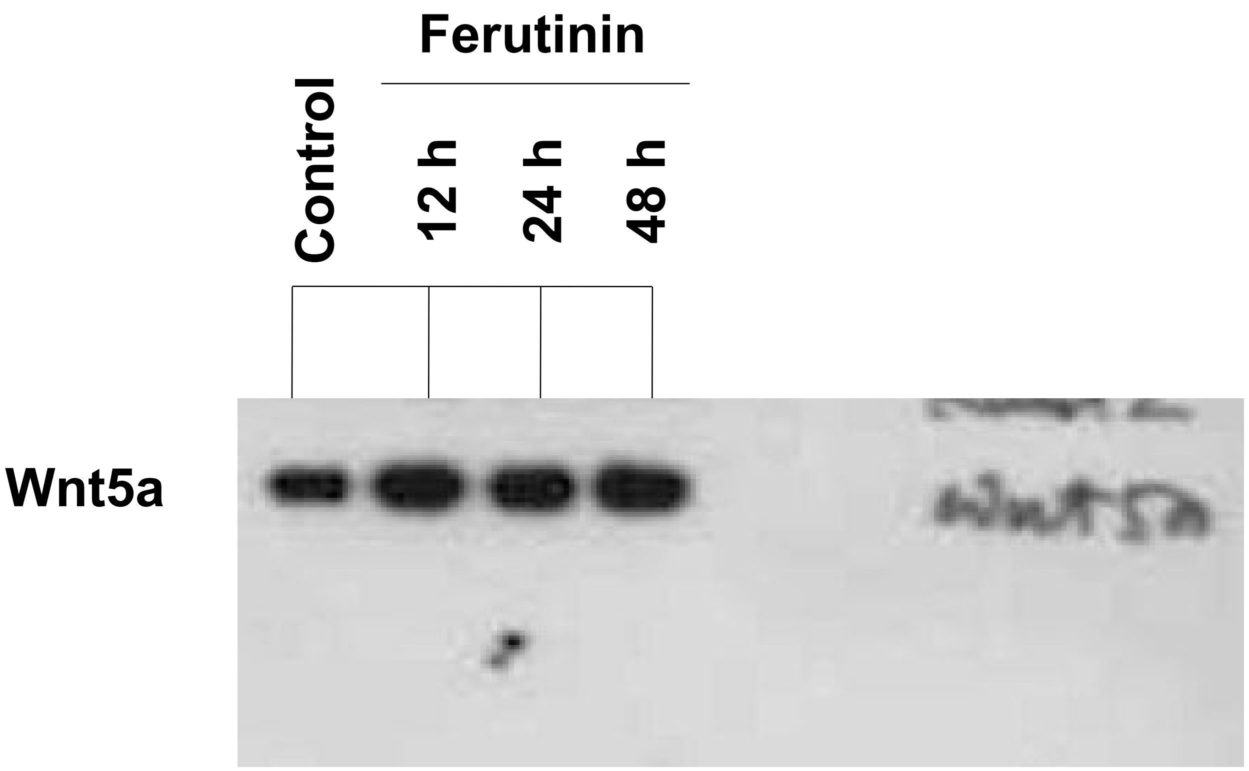

Figure 1

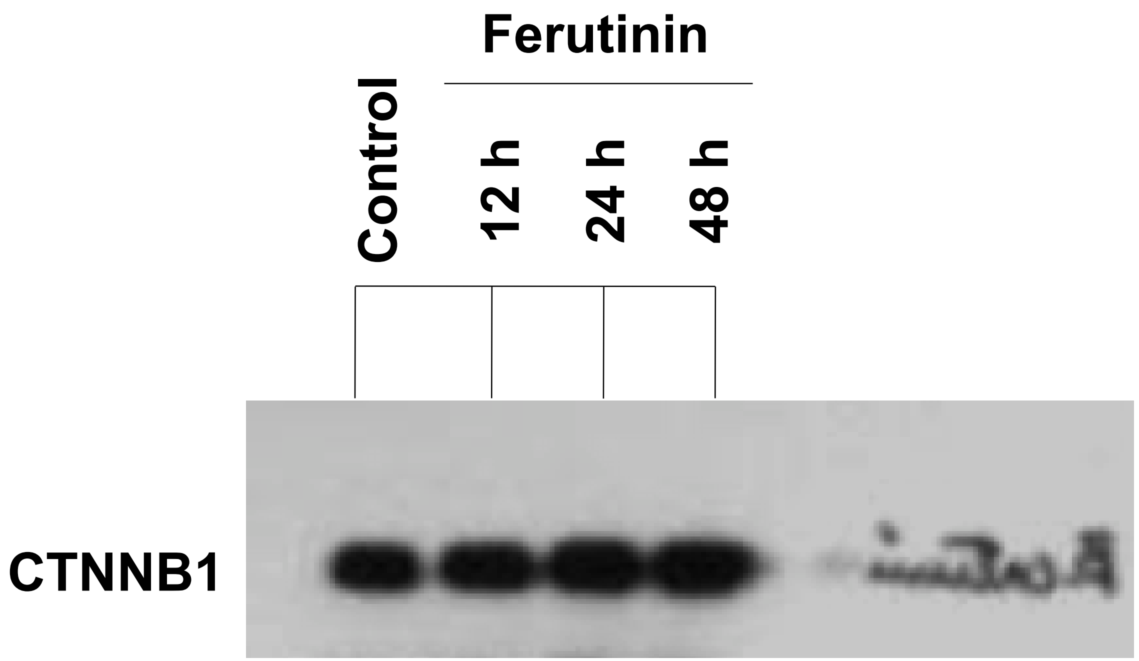

Figure 1

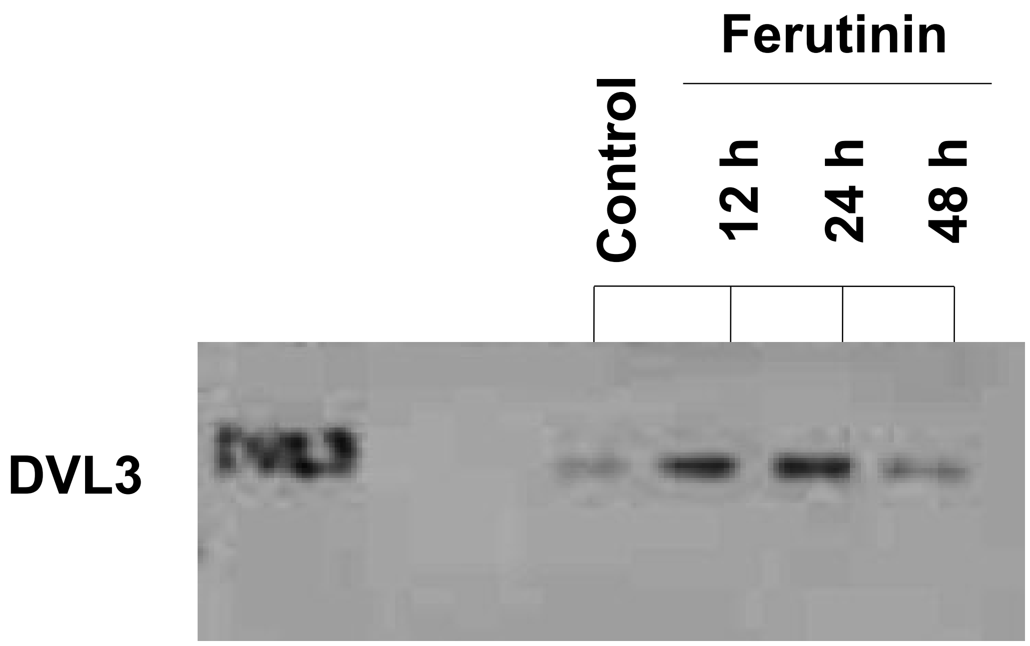

Figure 1

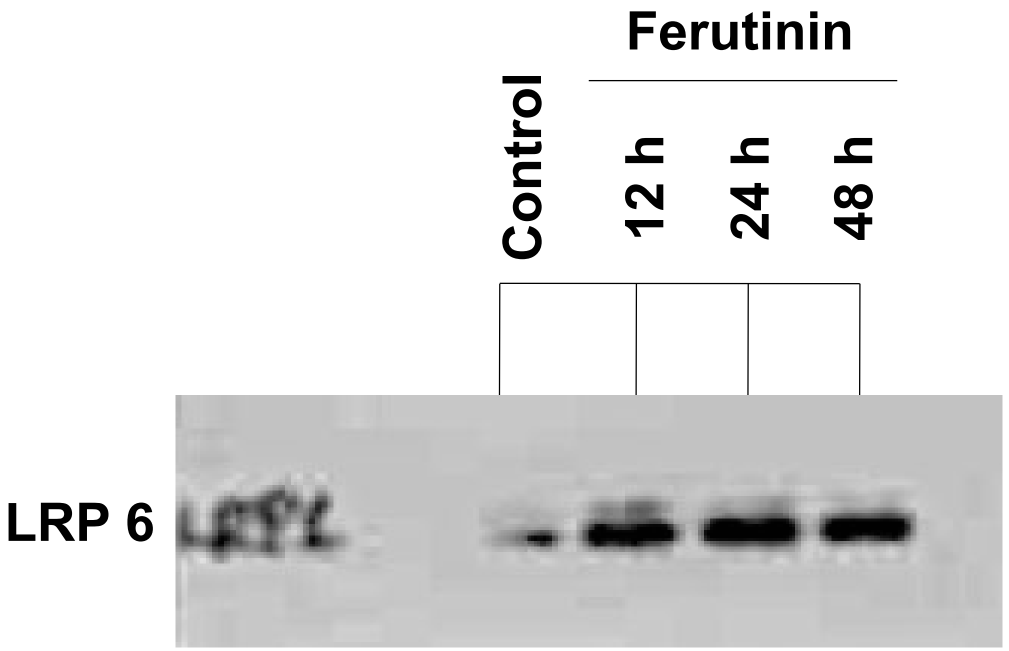

Figure 1

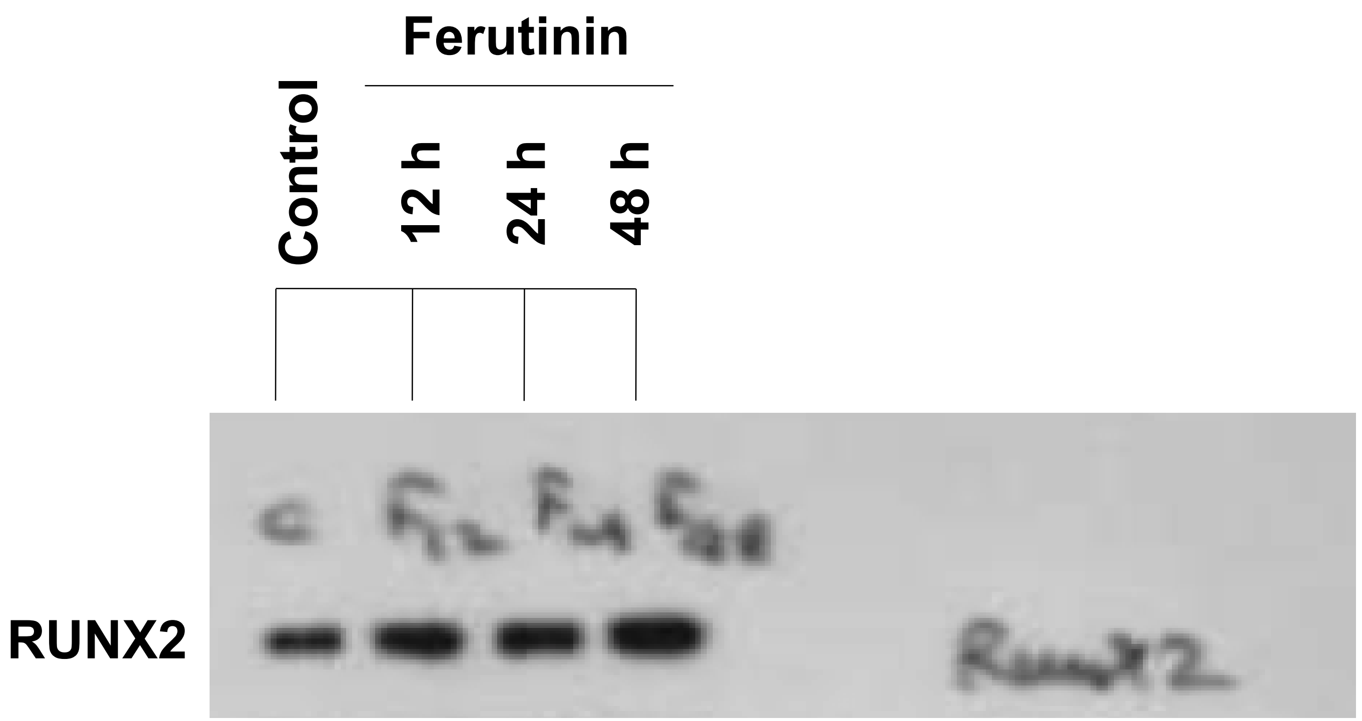

Figure 1

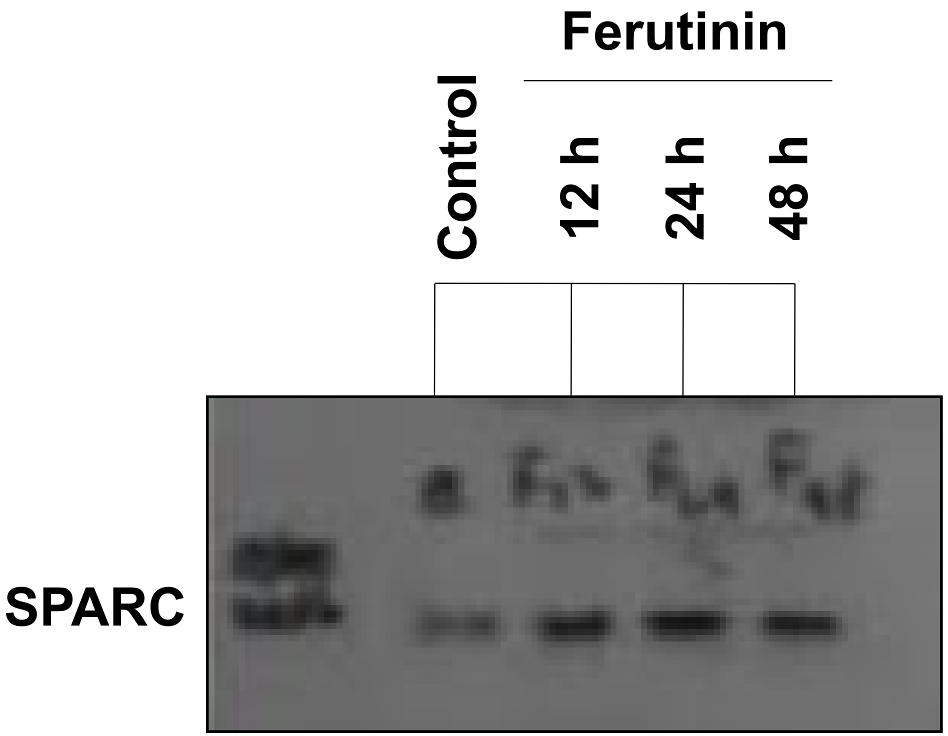

Figure 1

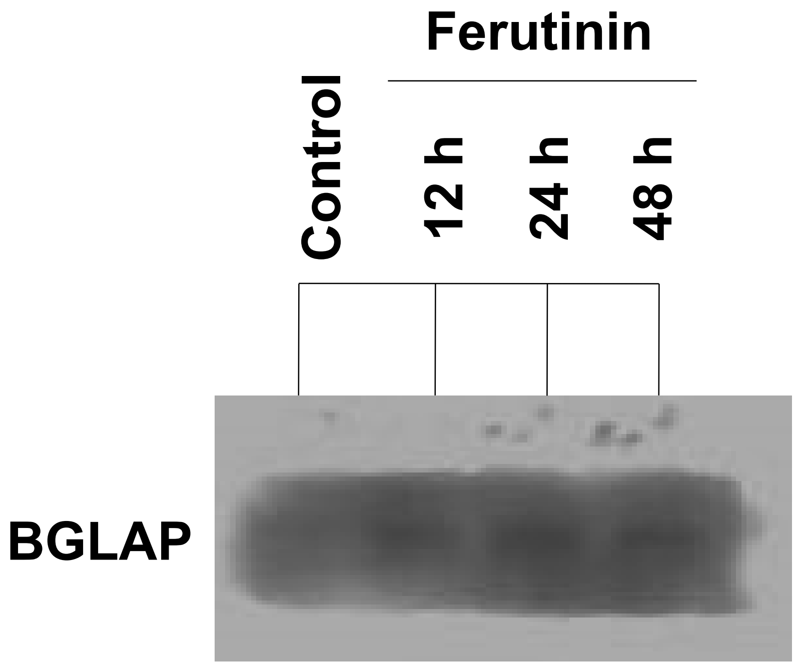

Figure 1

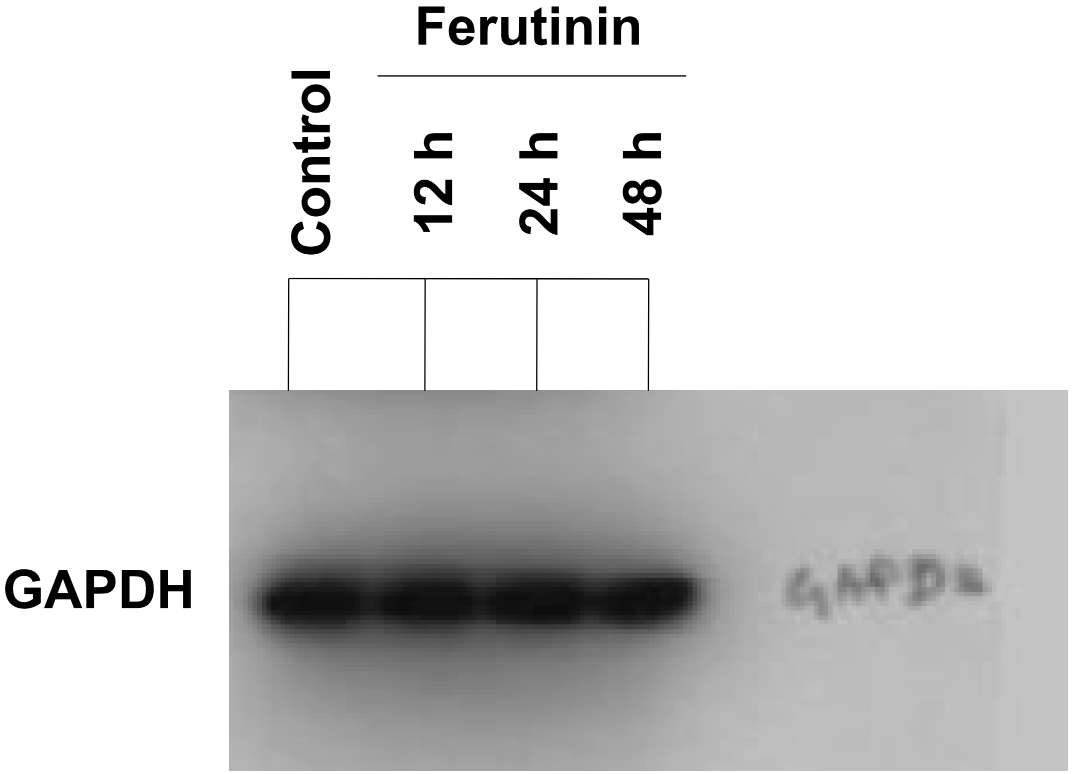

Figure 1

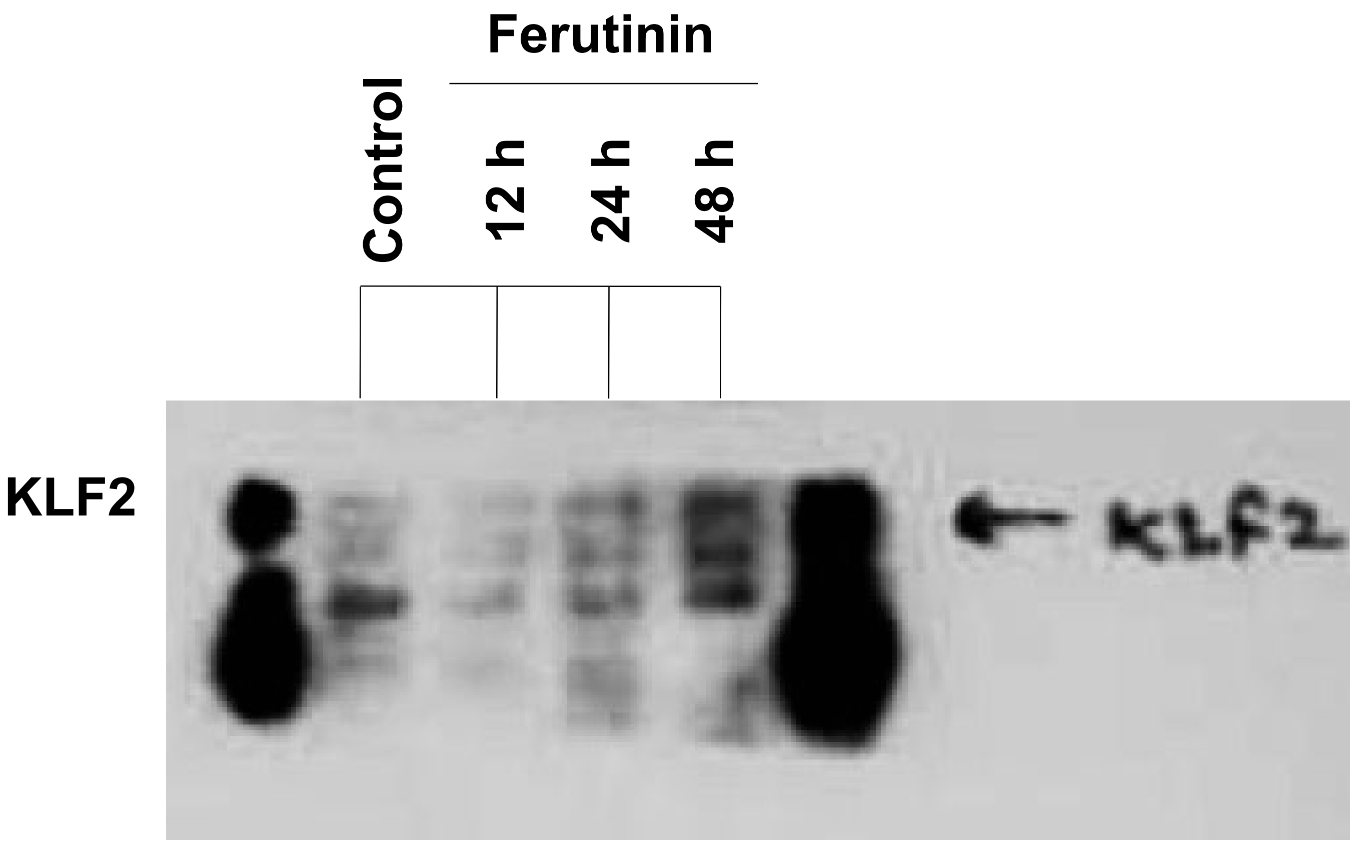

Figure 2B

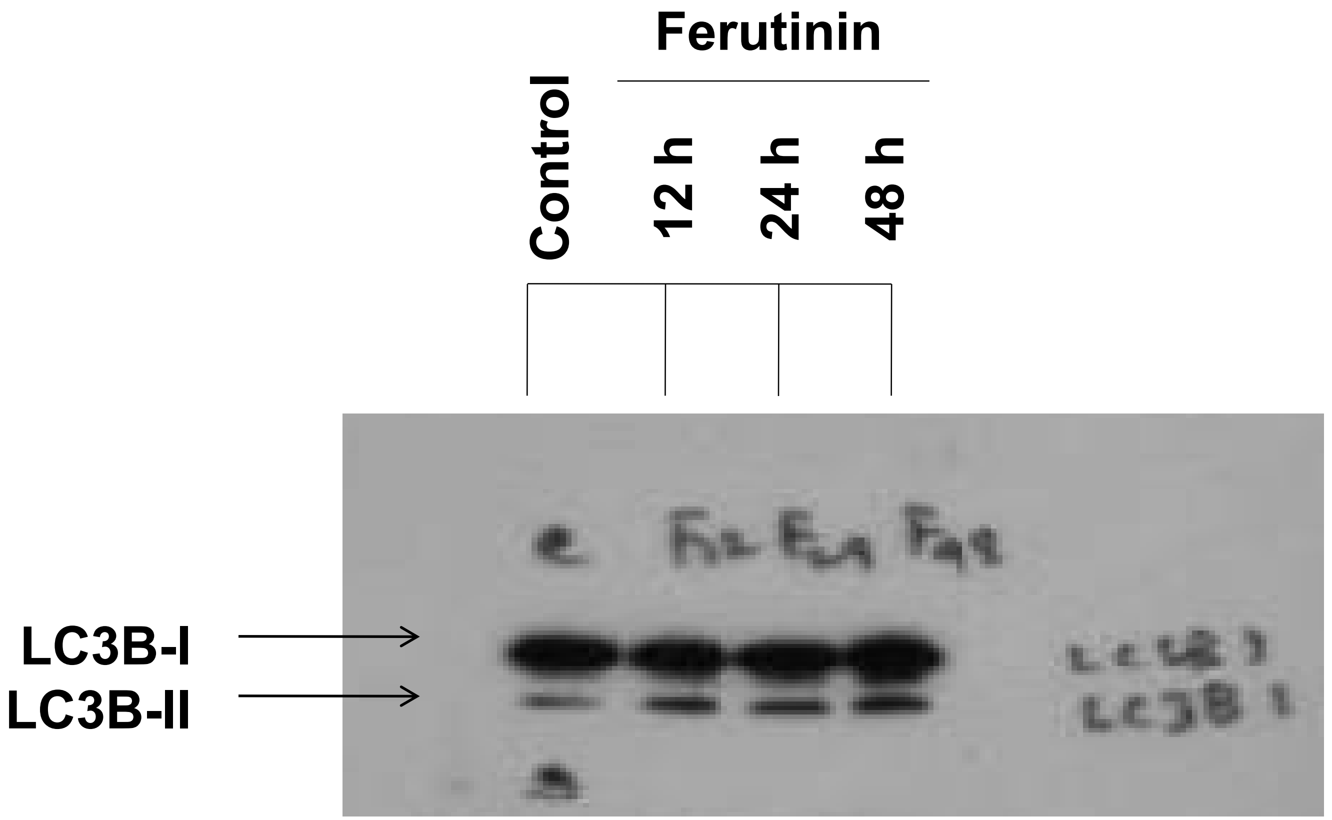

Figure 2B

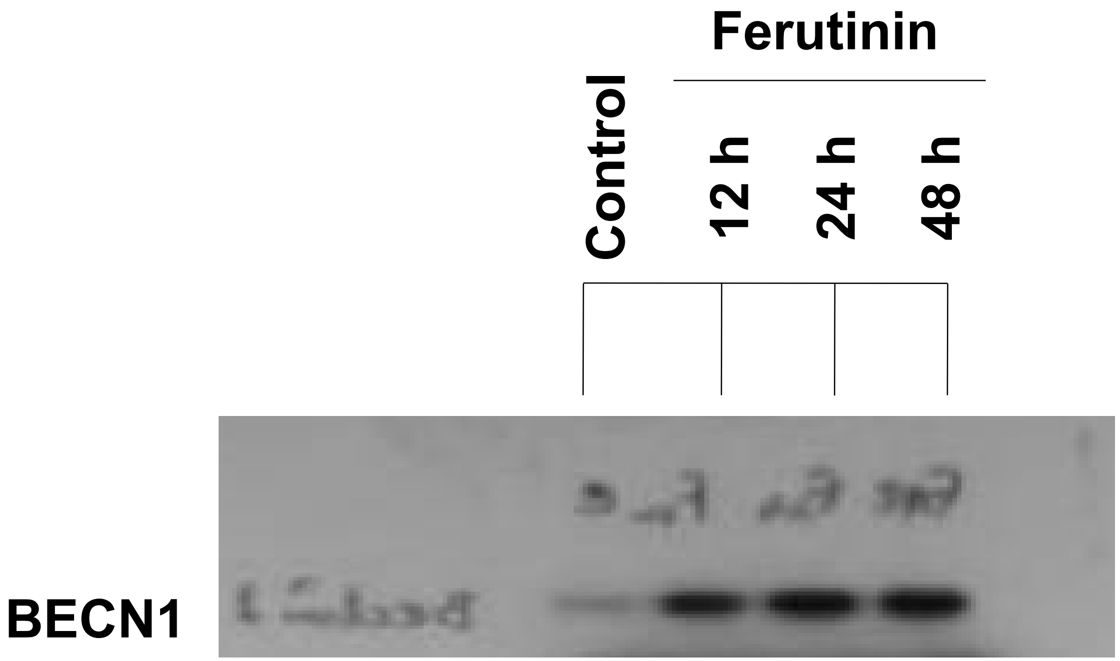

Figure 2B

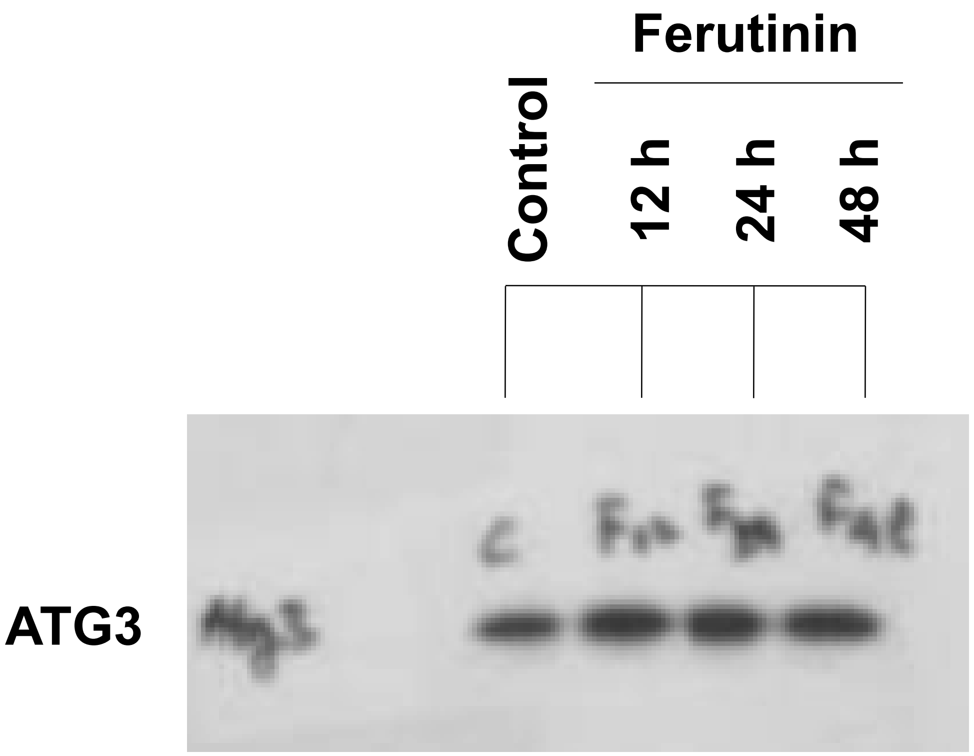

Figure 2B

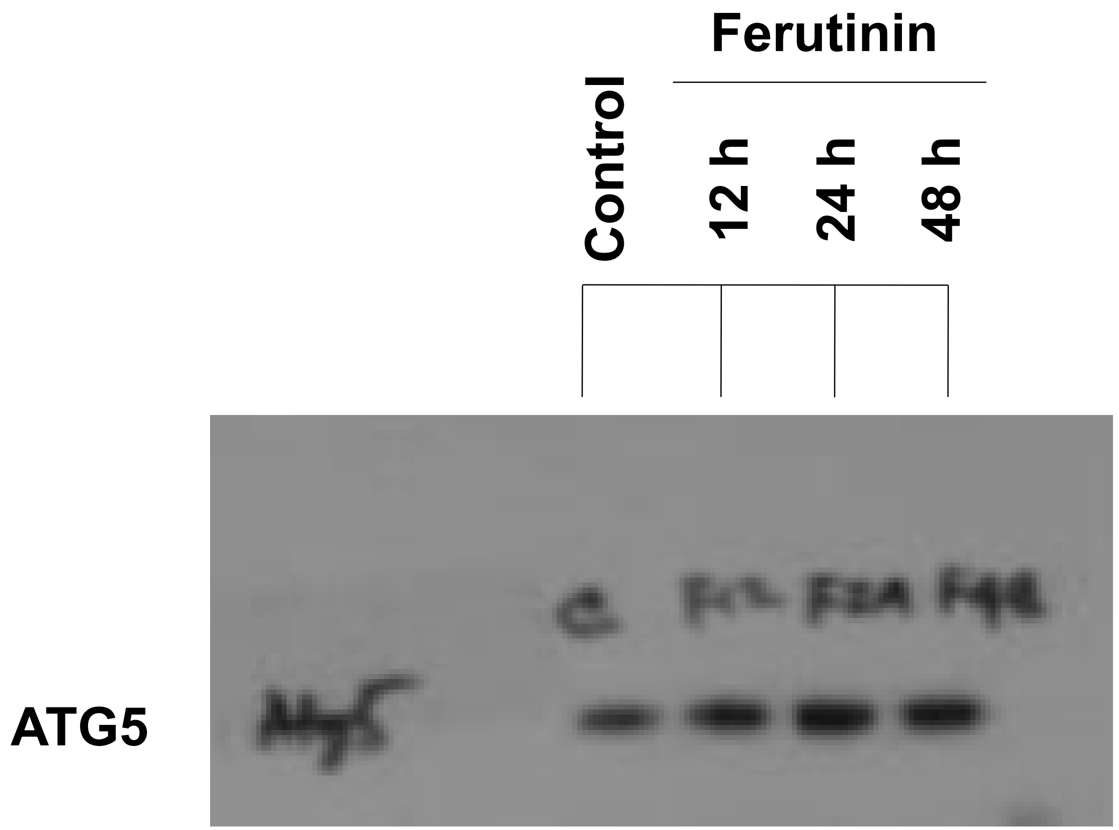

Figure 2B

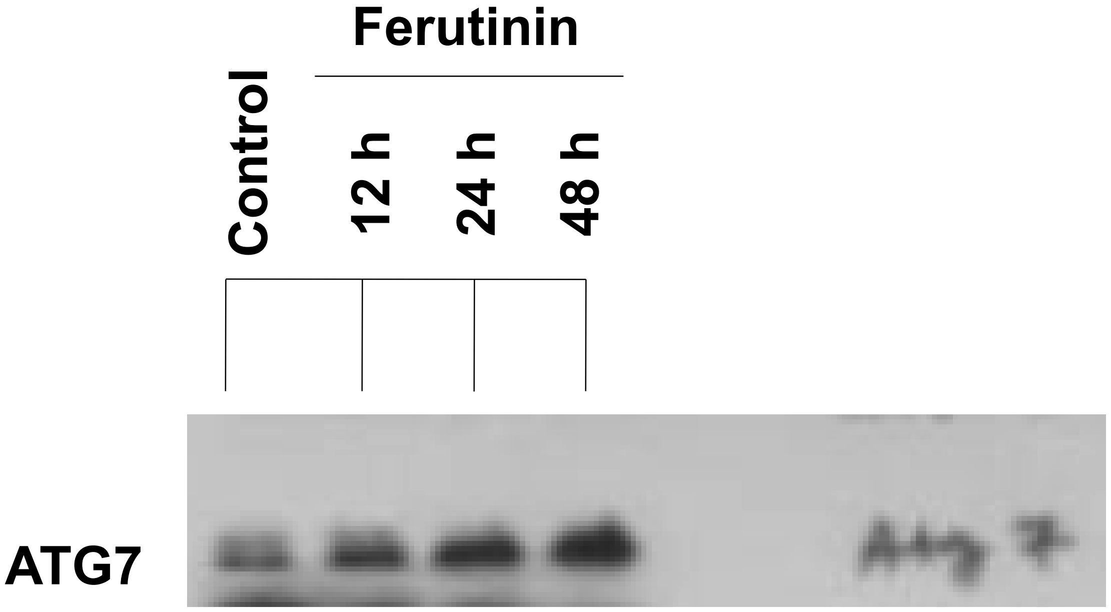

Figure 2B

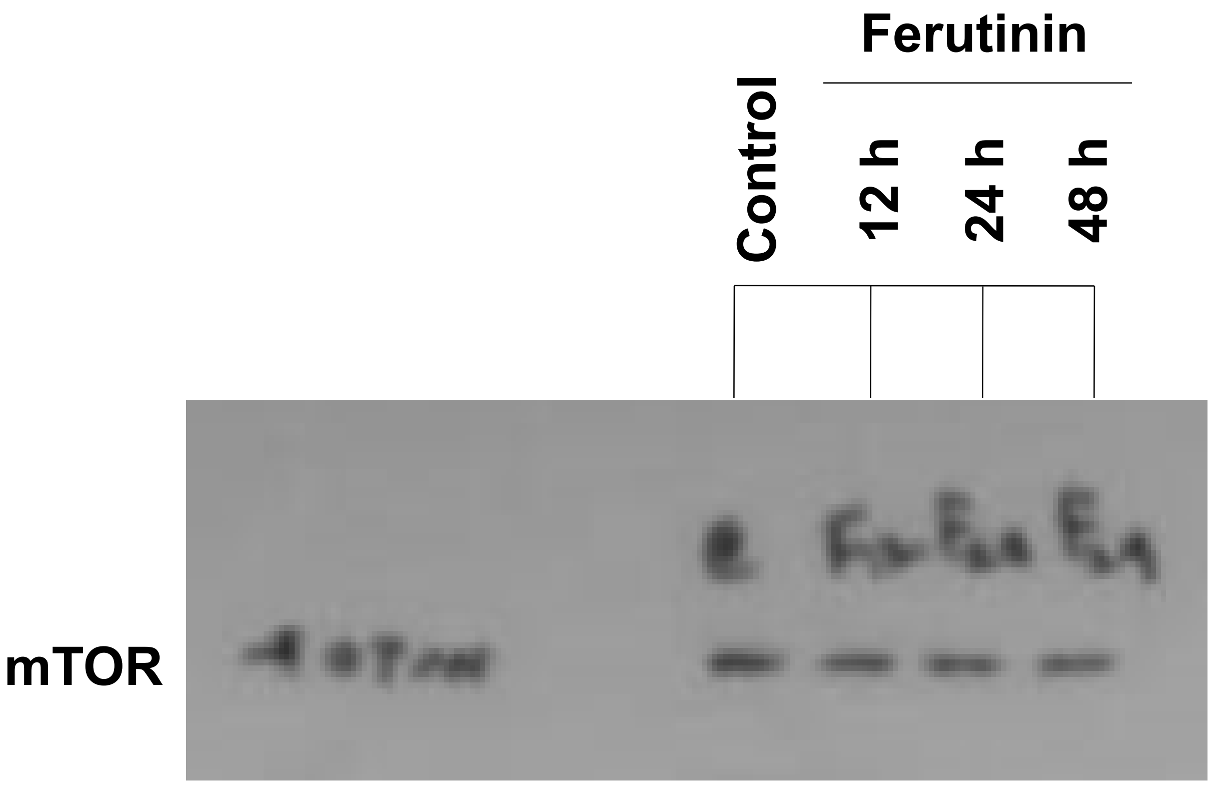

Figure 2B

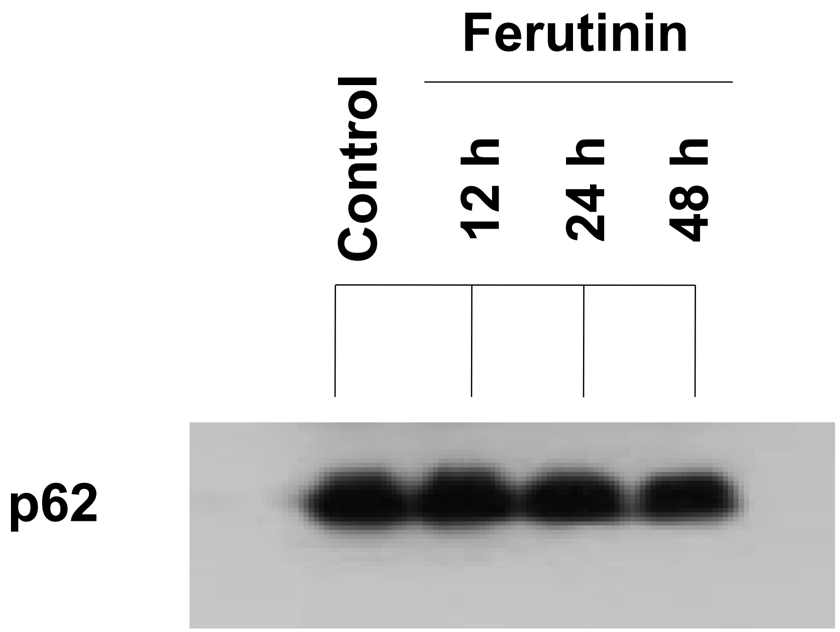

Figure 2B

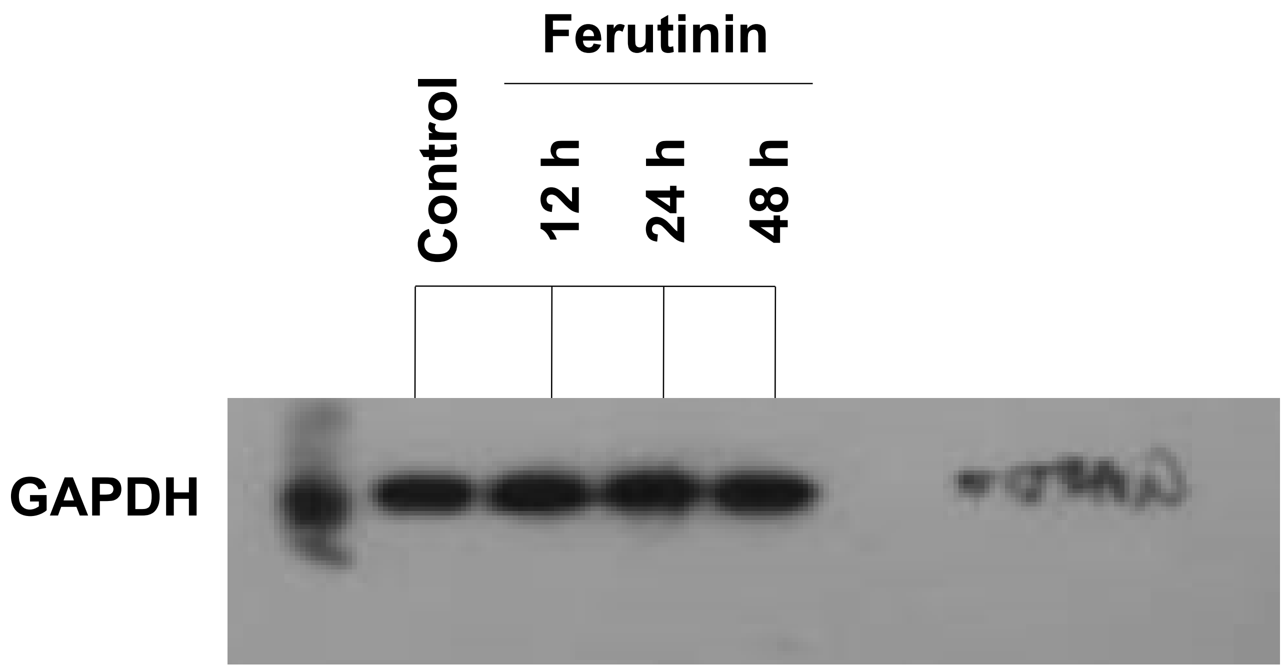

Figure 2B

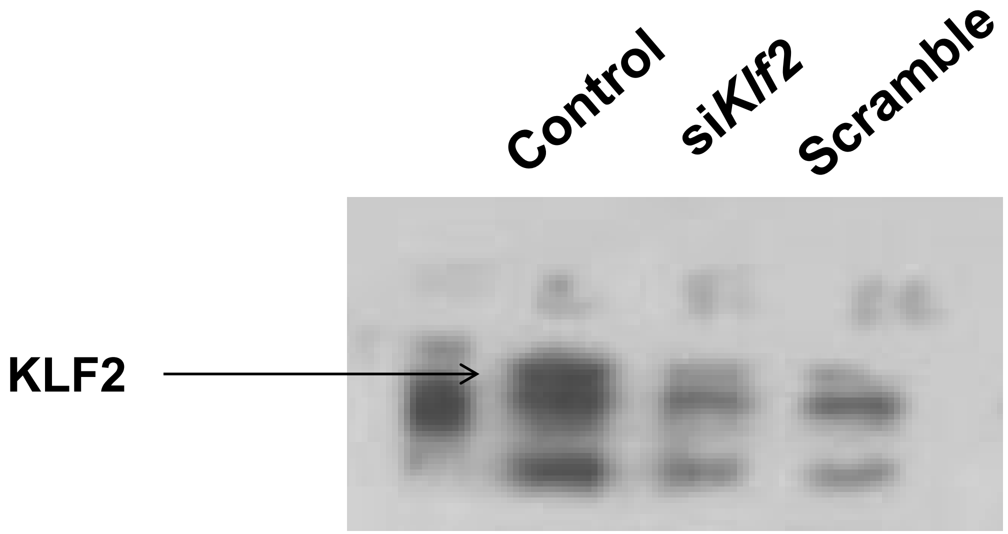

Figure 3A

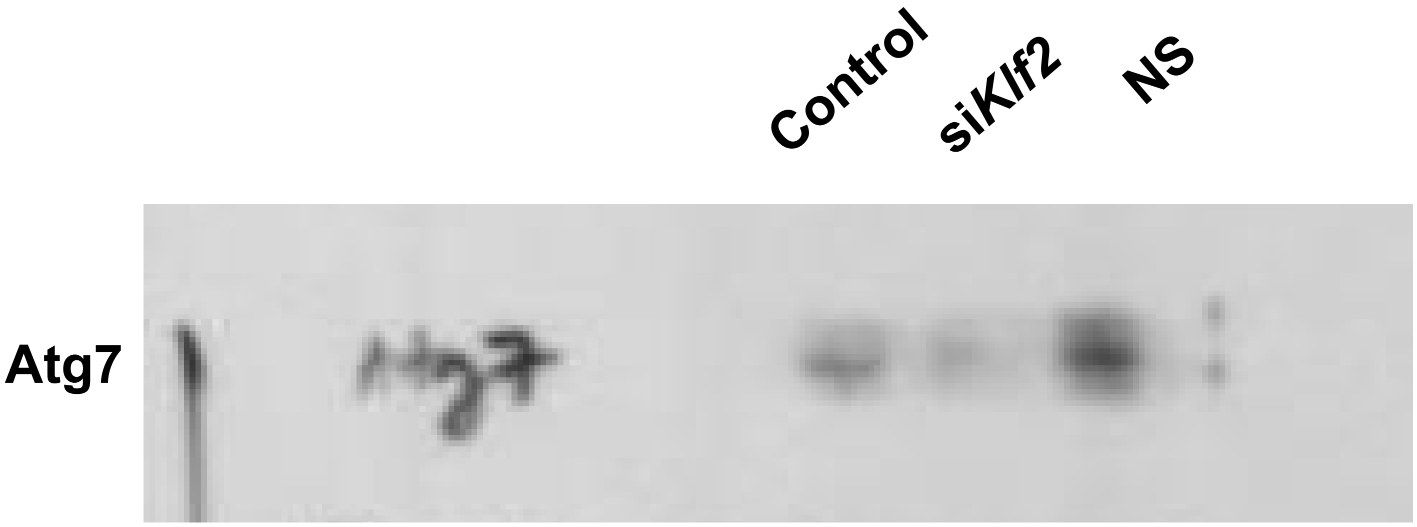

Figure 3A

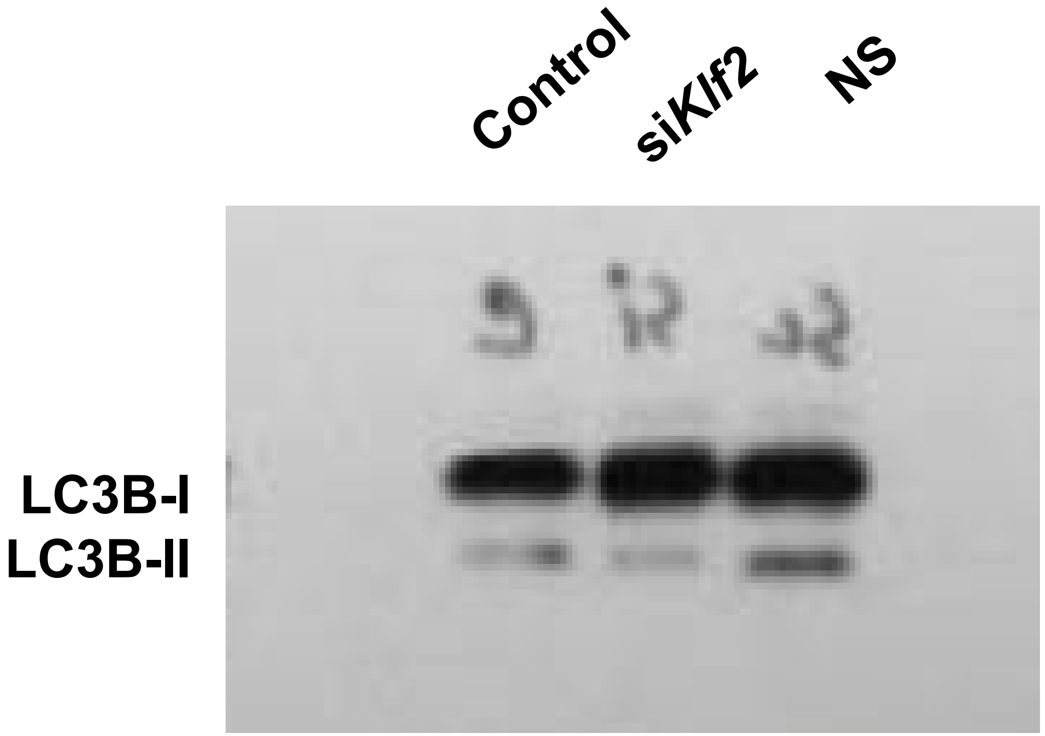

Figure 3A

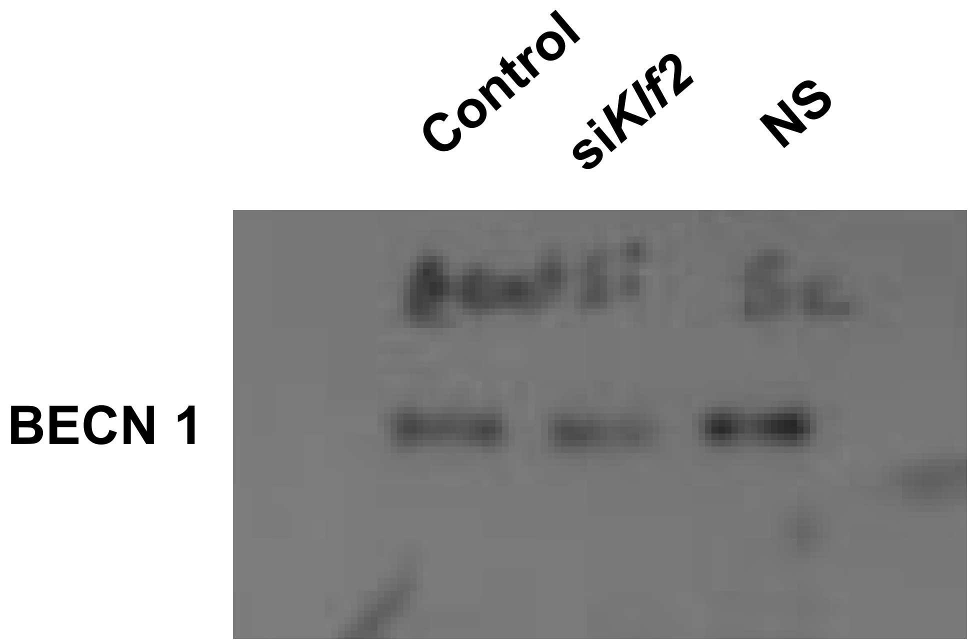

Figure 3A

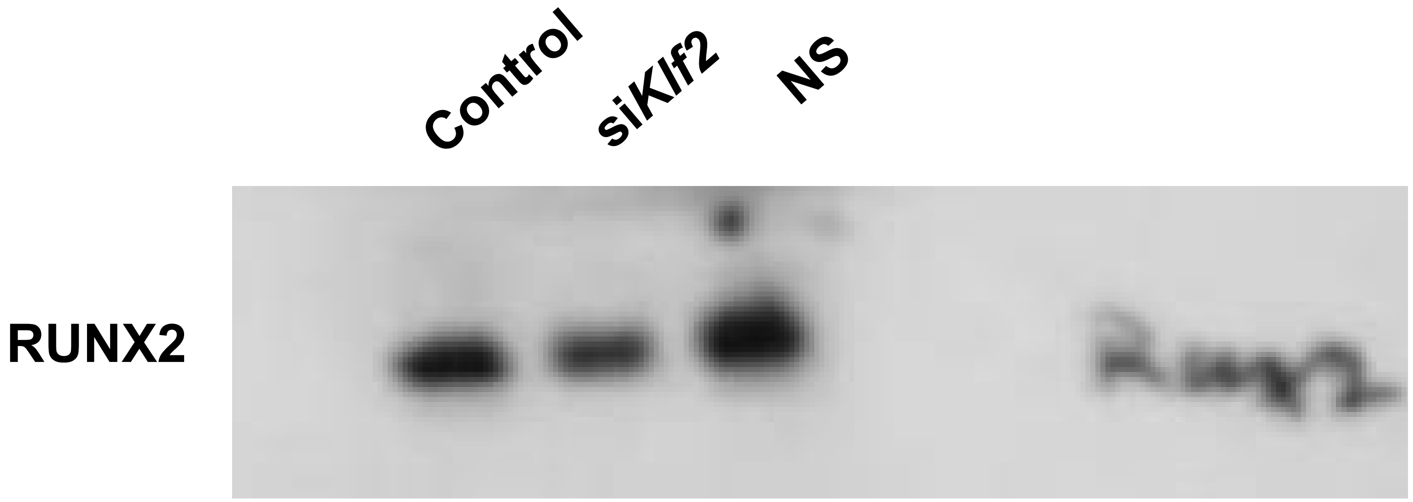

Figure 3A

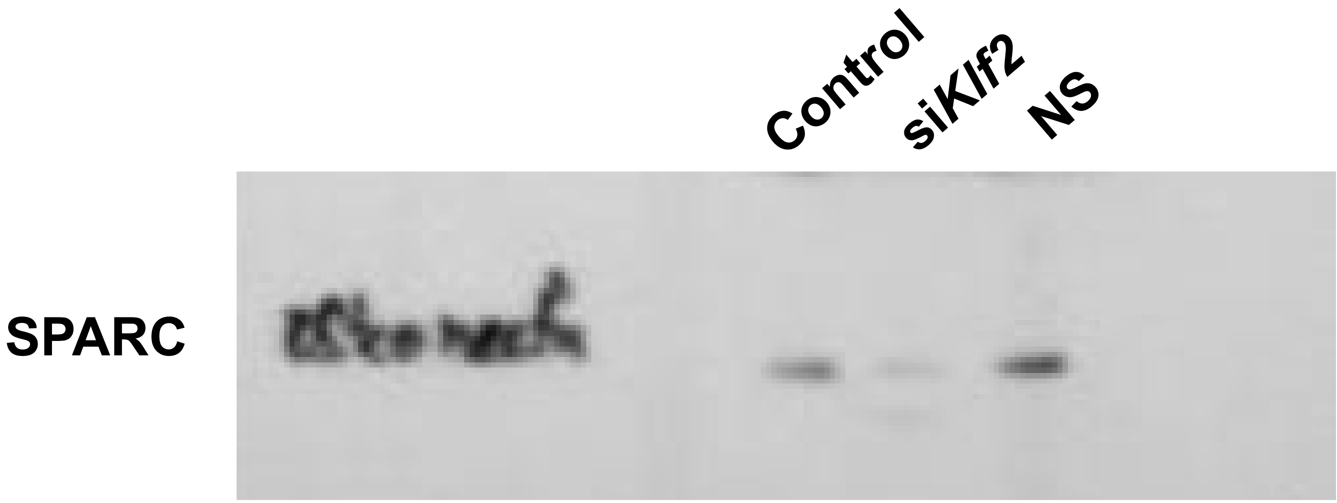

Figure 3A

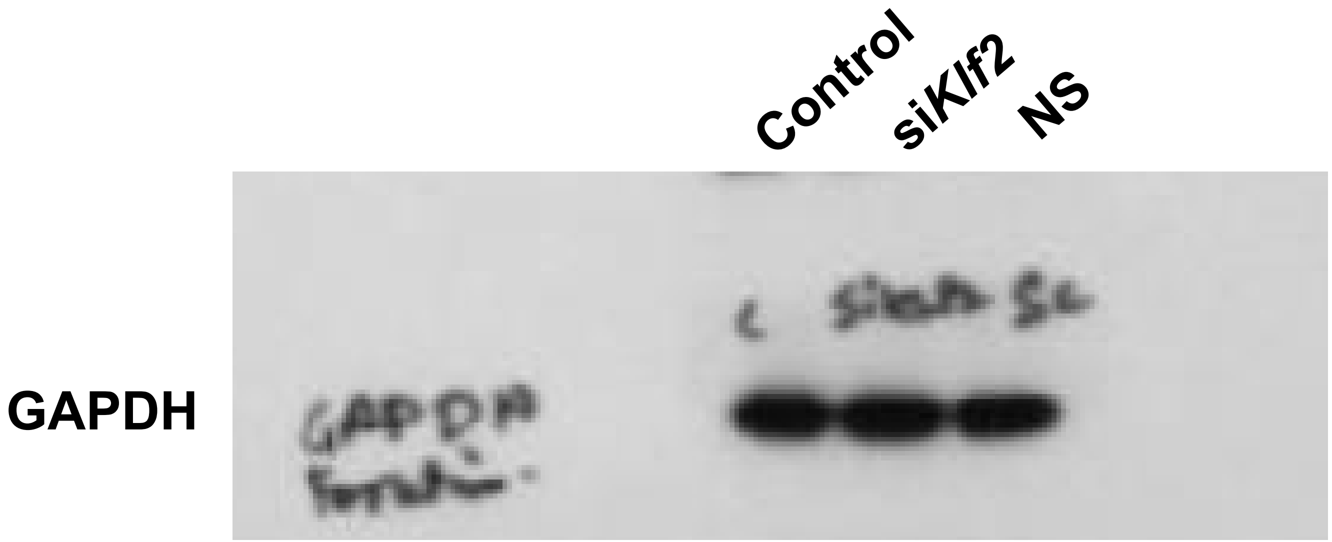

Figure 3A

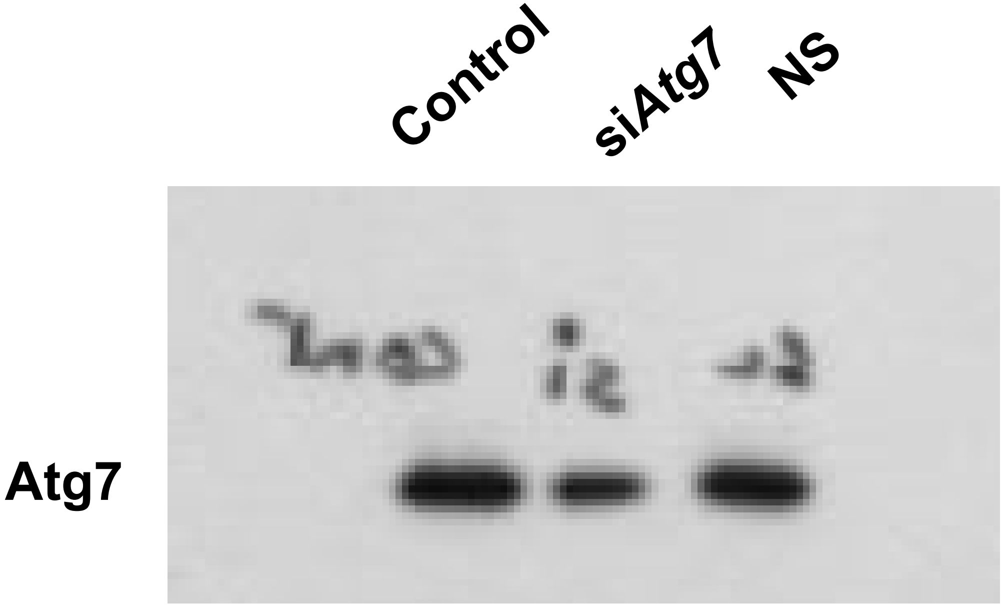

Figure 3B

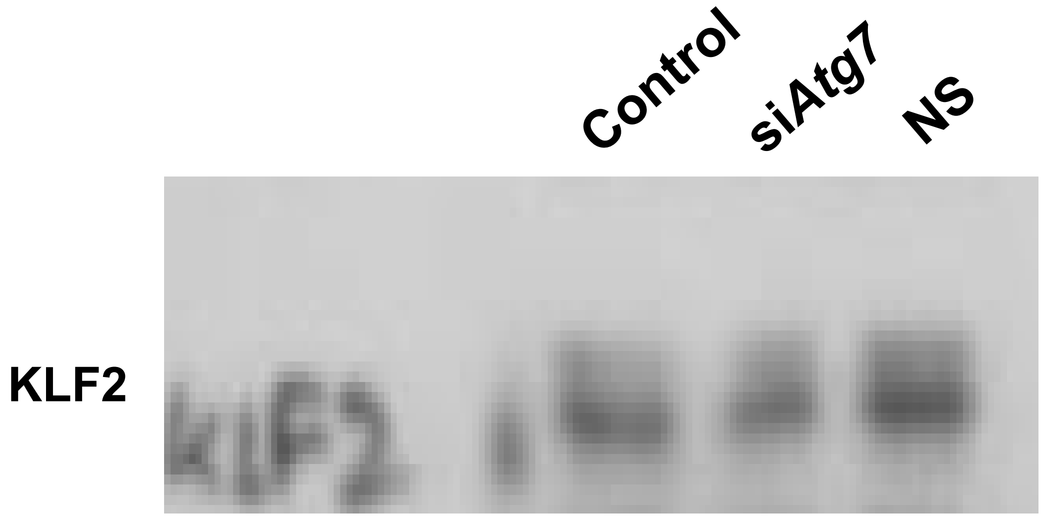

Figure 3B

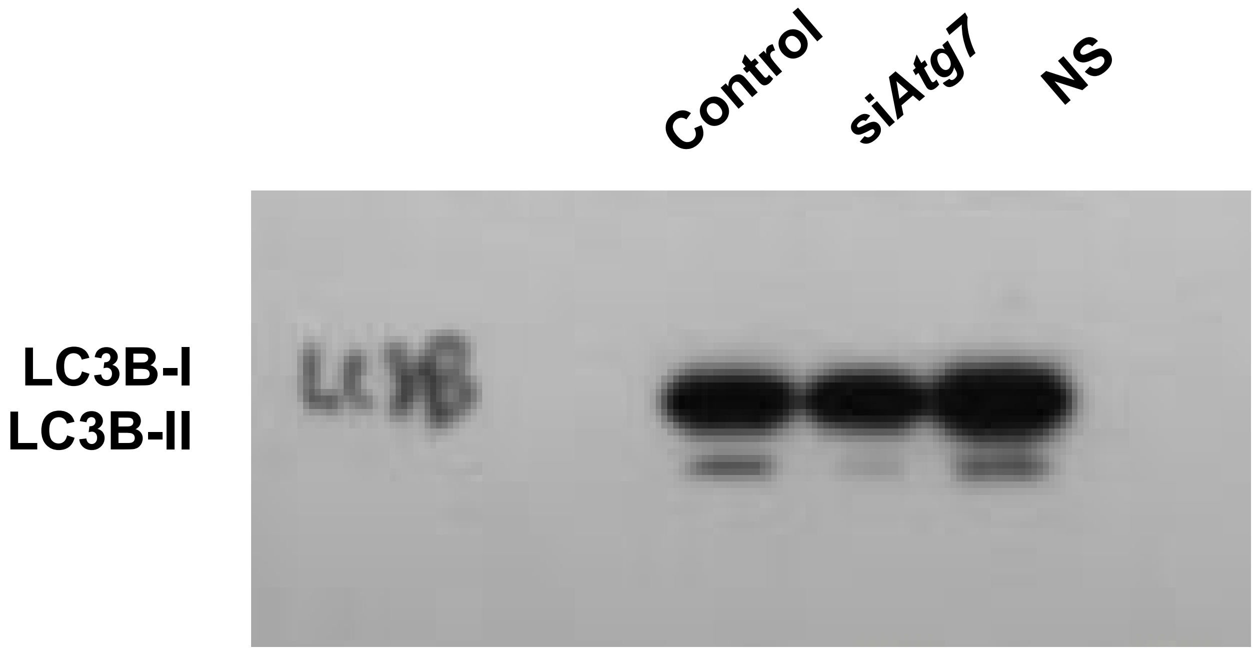

Figure 3B

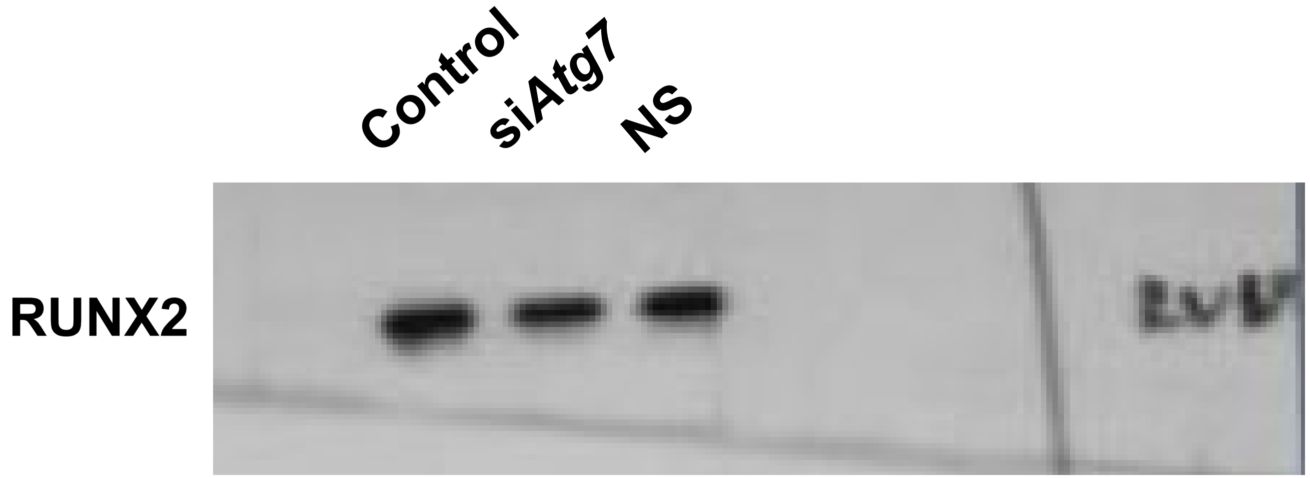

Figure 3B

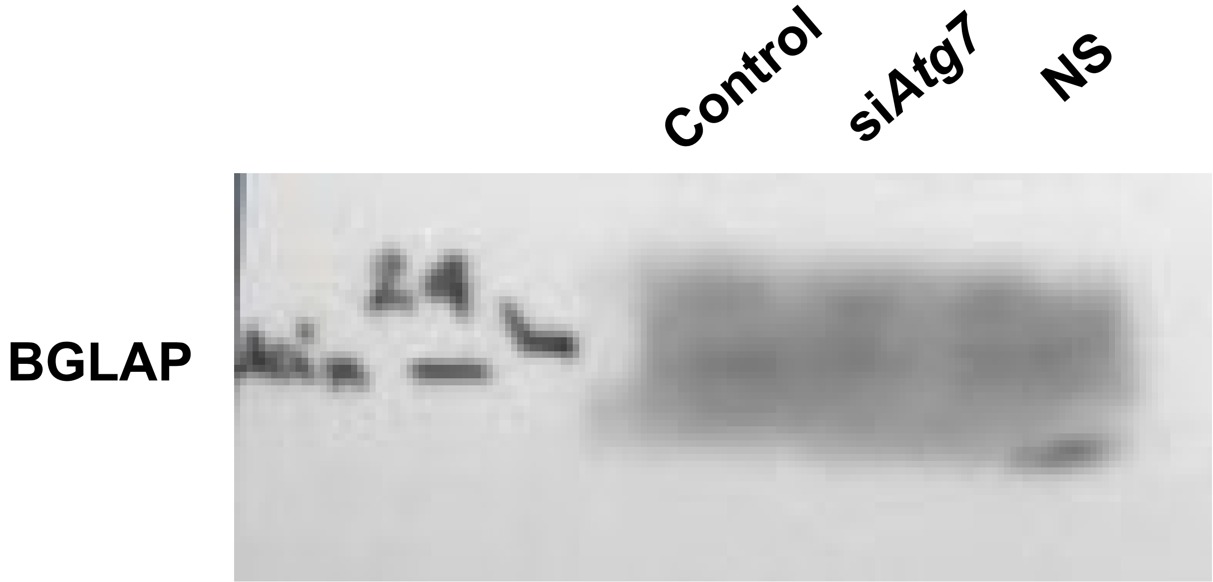

Figure 3B

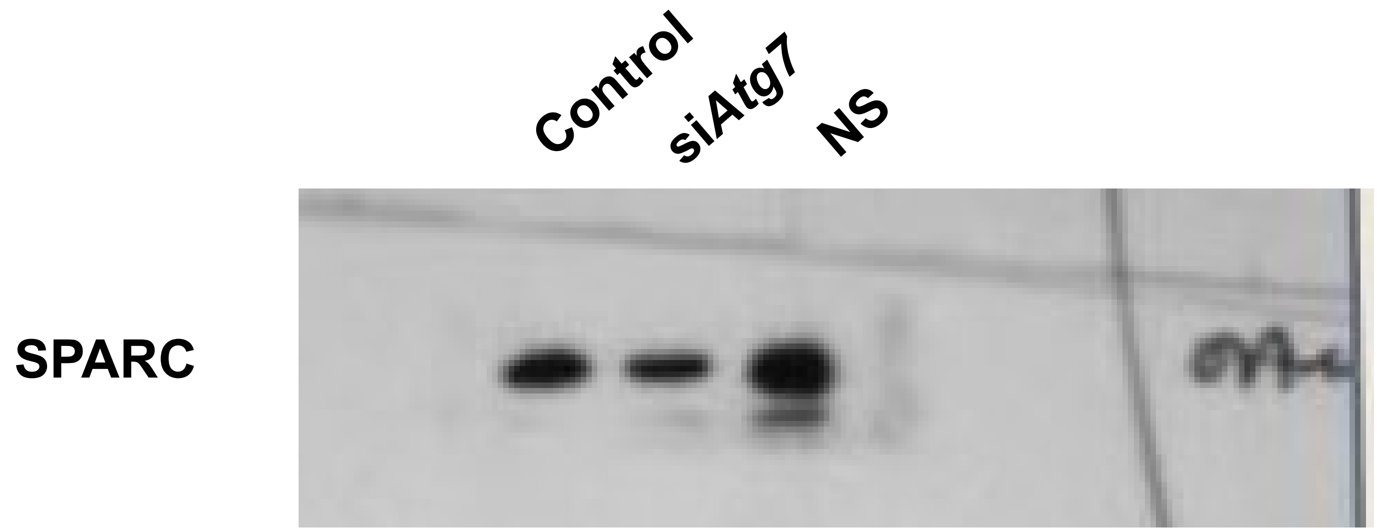

Figure 3B

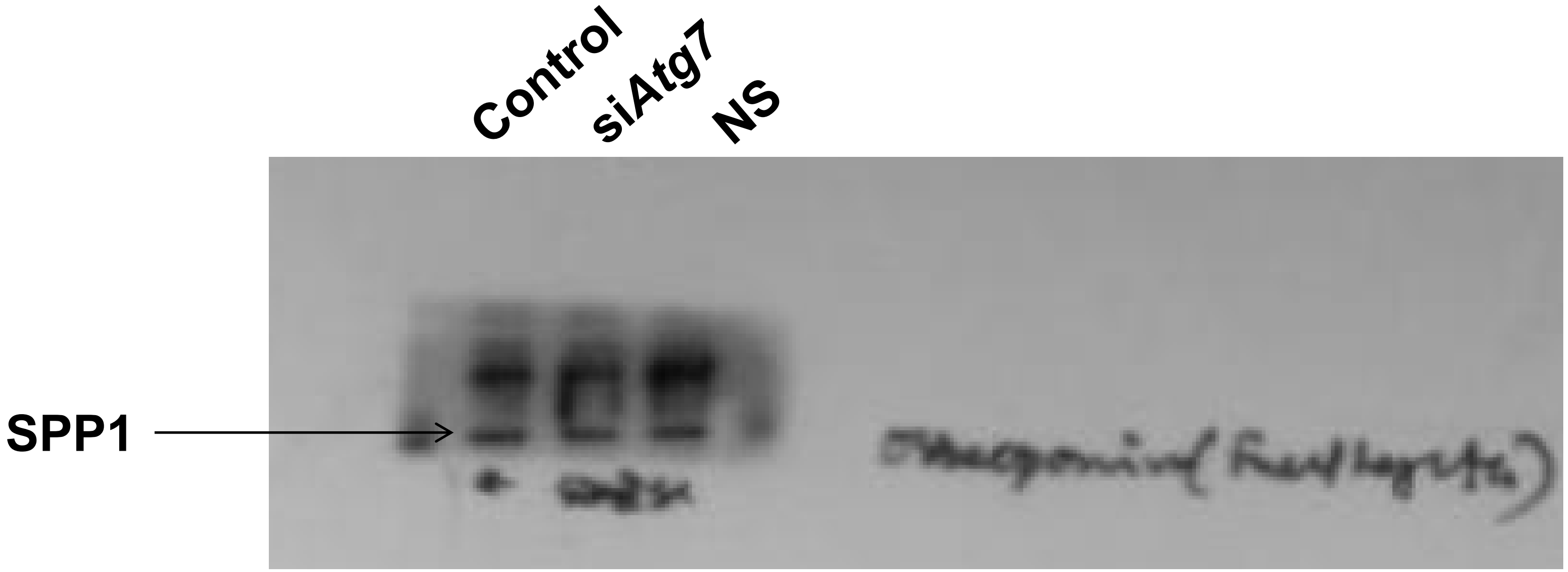

Figure 3B

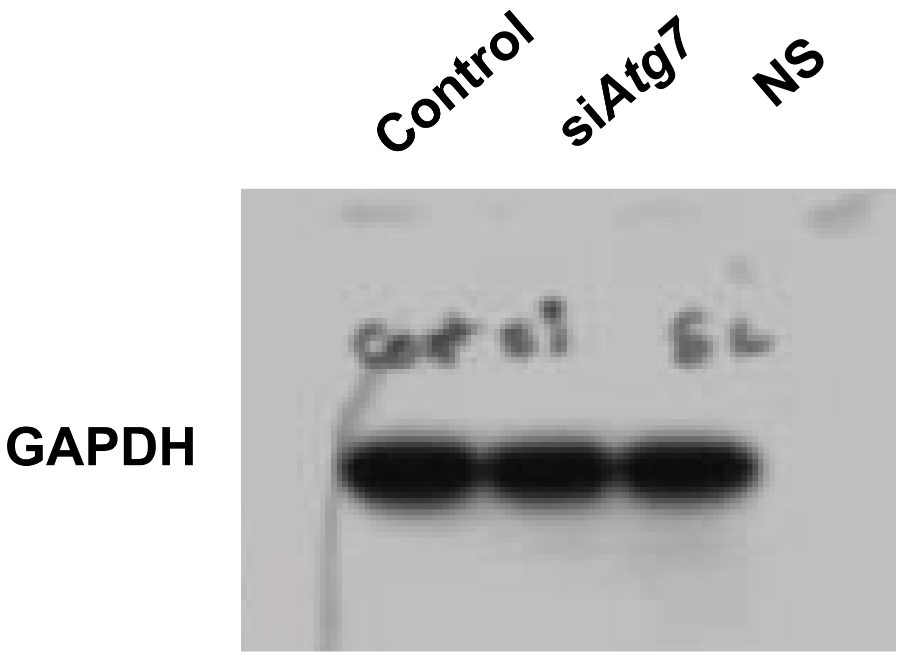

Figure 3B

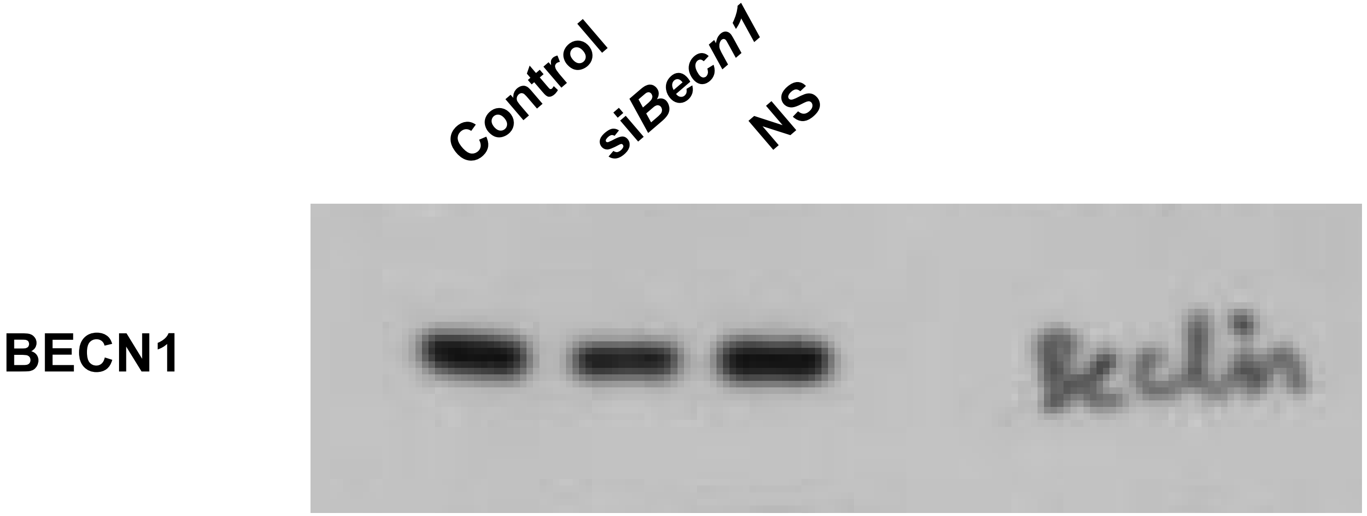

Figure 3C

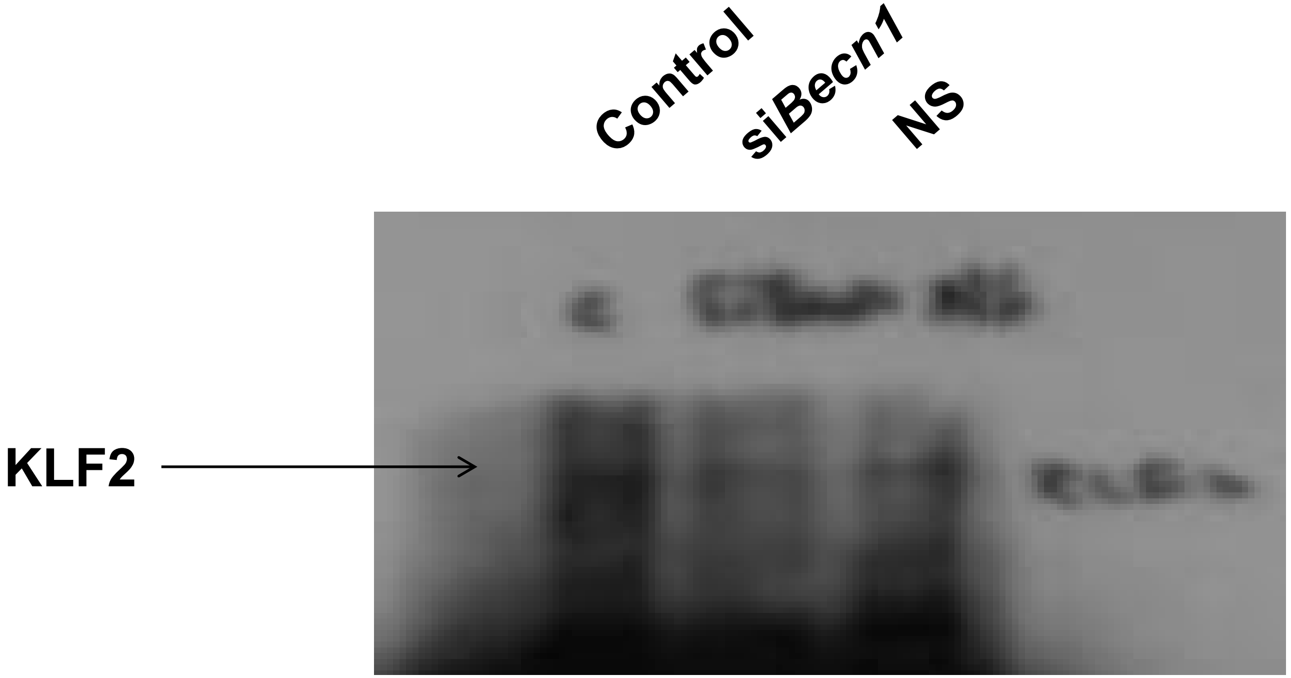

Figure 3C

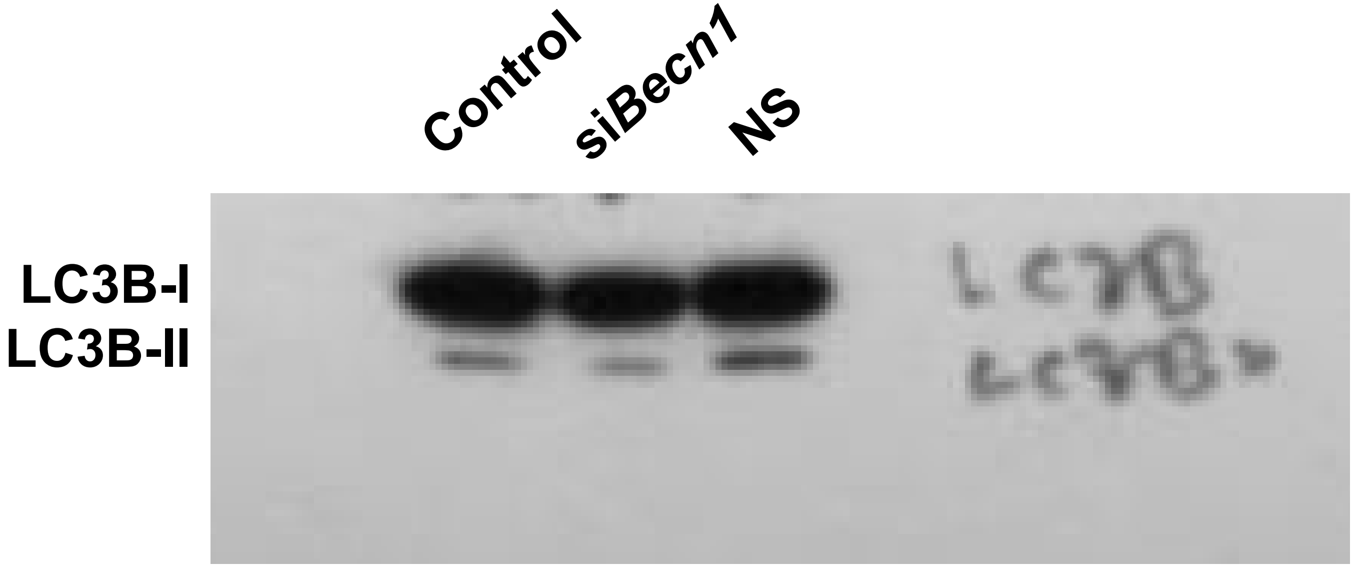

Figure 3C

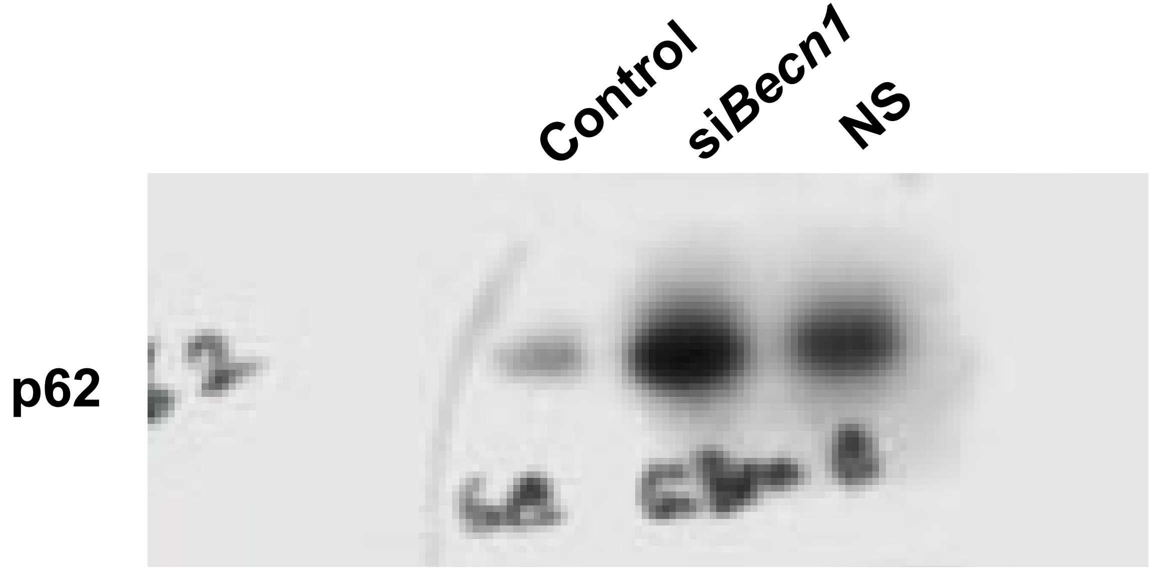

Figure 3C

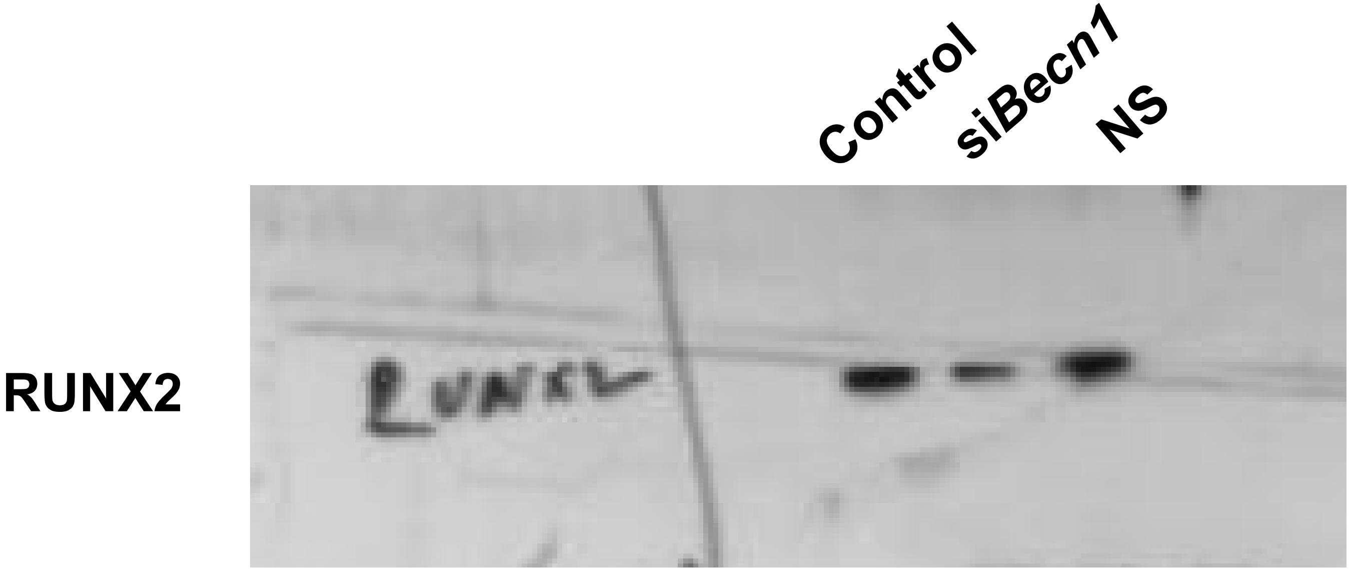

Figure 3C

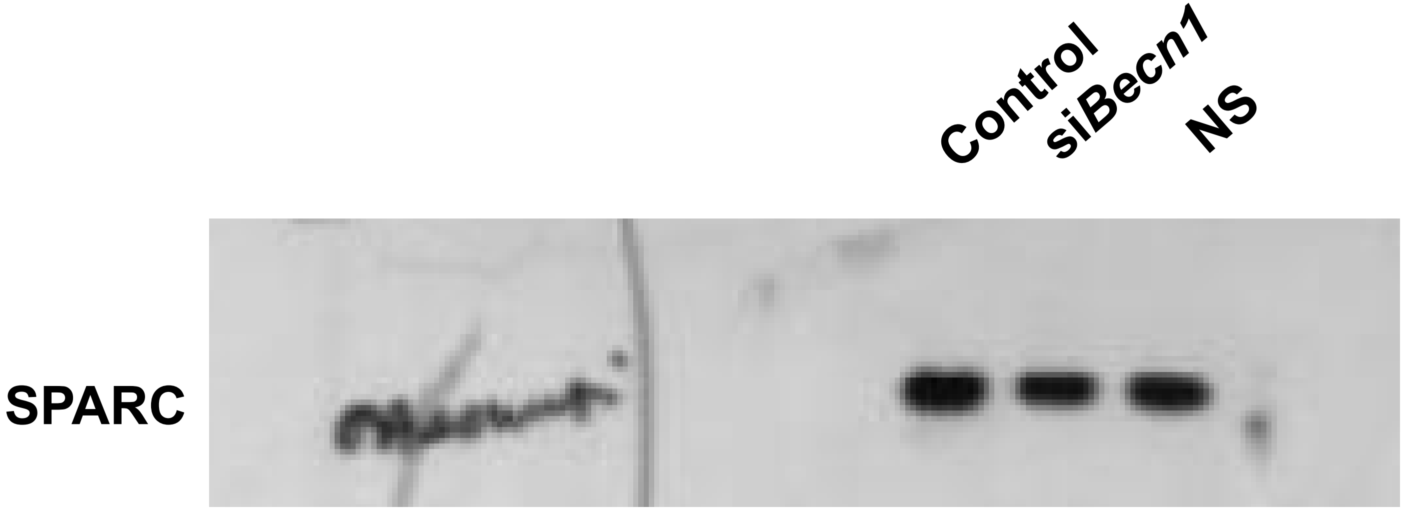

Figure 3C

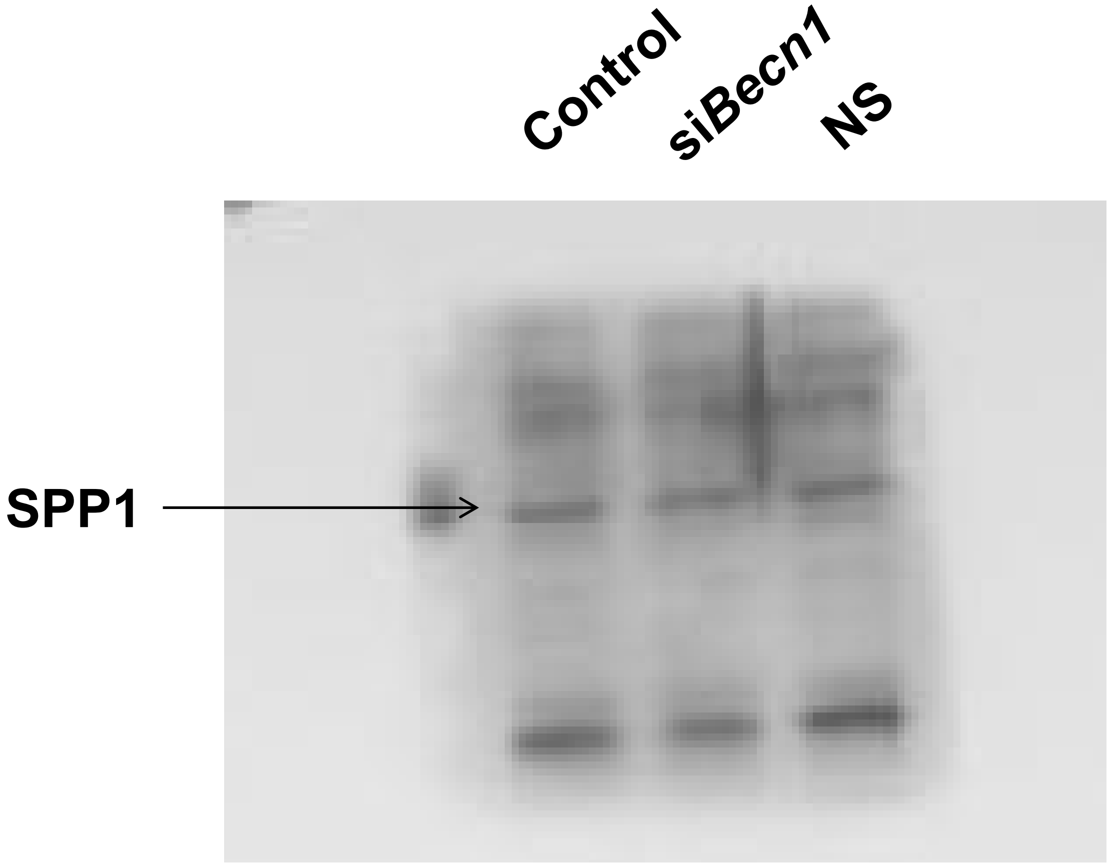

Figure 3C

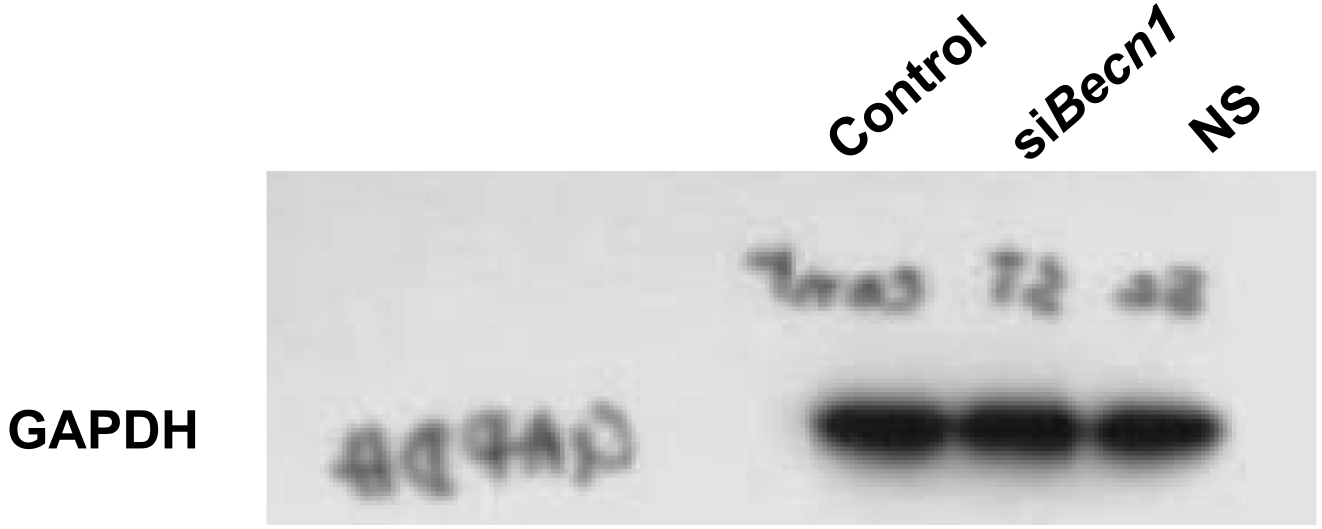

Figure 3C

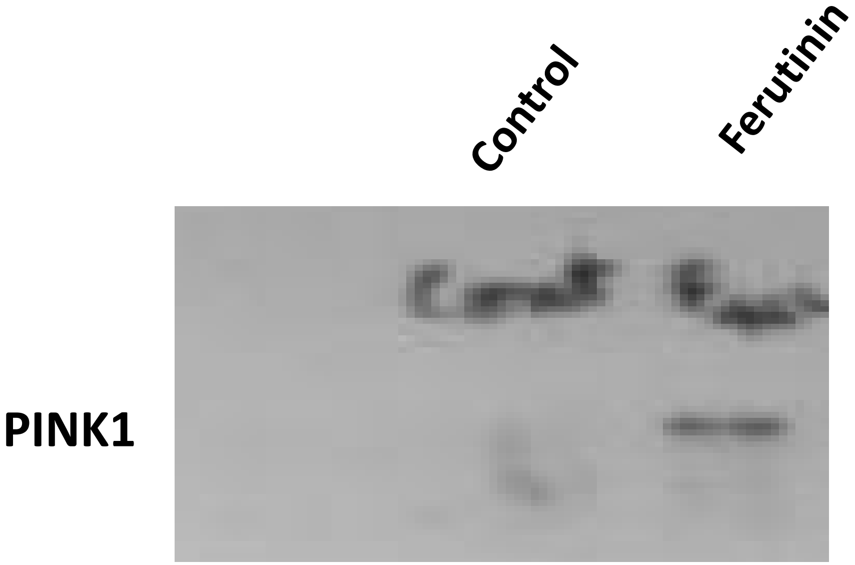

Figure 5B

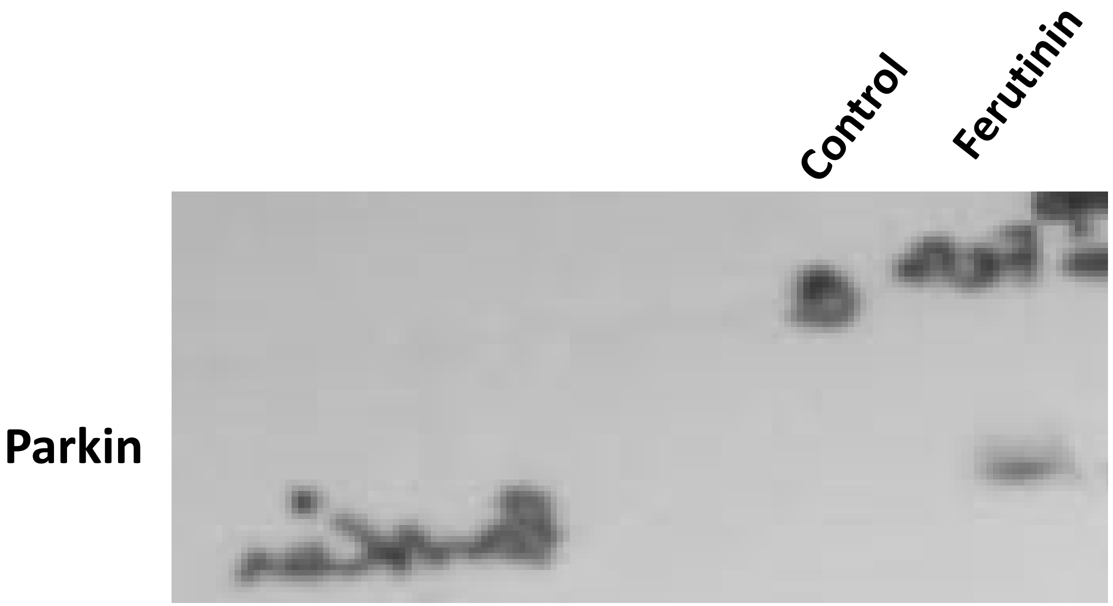

Figure 5B

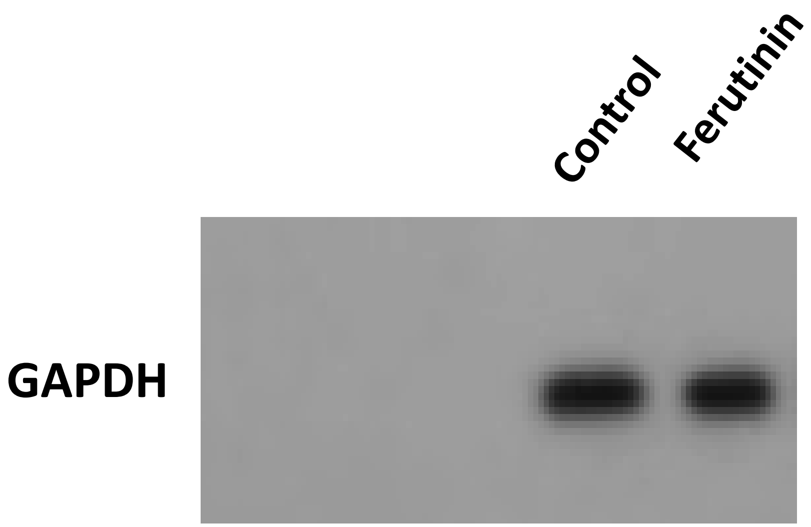

Figure 5B
