# Supplementary material for: Ferutinin induces osteoblast differentiation of DPSCs via induction of KLF2 and autophagy/mitophagy
Source: Cell Death Dis. 2022 May 12;13(5):452. doi: 10.1038/s41419-022-04903-9 (PMC9098908; doi:10.1038/s41419-022-04903-9)
Supplement: Supplementary file 4 — Supplemental Figures [file 41419_2022_4903_MOESM4_ESM.pdf]

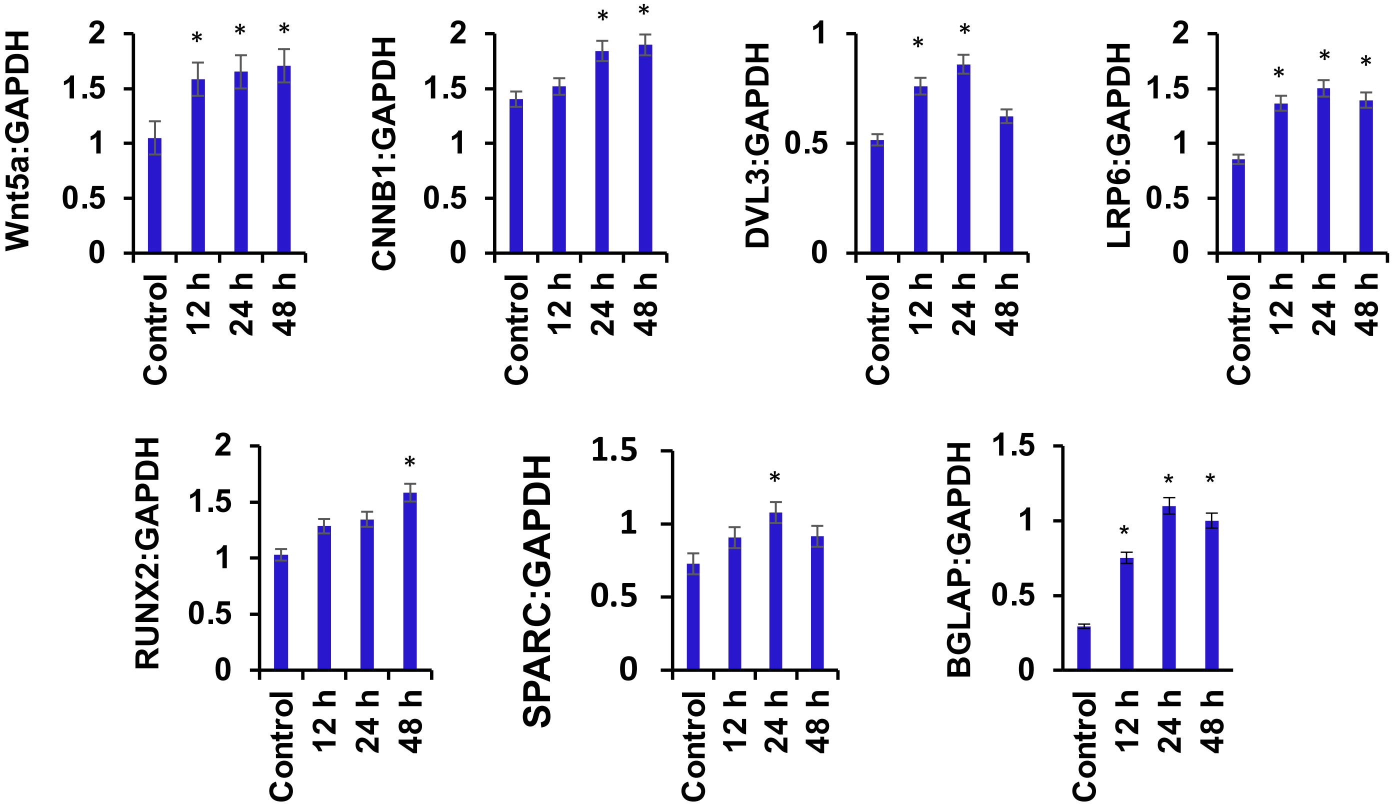

Wnt5a:GAPDH
CNNB1:GAPDH
DVL3:GAPDH
LRP6:GAPDH
RUNX2:GAPDH
SPARC:GAPDH
BGLAP:GAPDH
0
0.5
1
1.5
2
Control
12 h
24 h
48 h
*

Figure S1

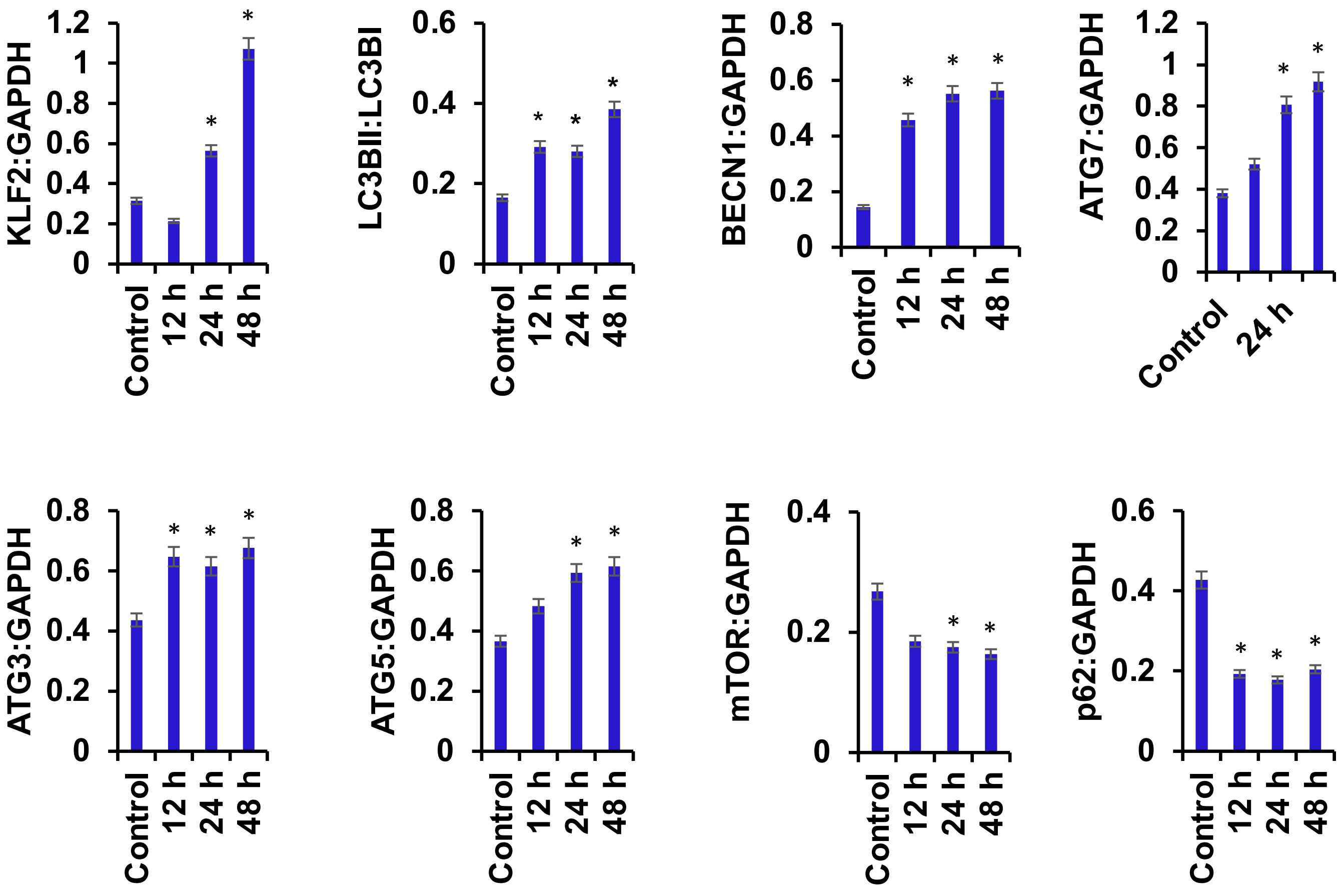
KLF2:GAPDH
LC3BII:LC3BI
BECN1:GAPDH
ATG7:GAPDH
ATG3:GAPDH
ATG5:GAPDH
mTOR:GAPDH
p62:GAPDH
Control
12 h
24 h
48 h

Figure S2

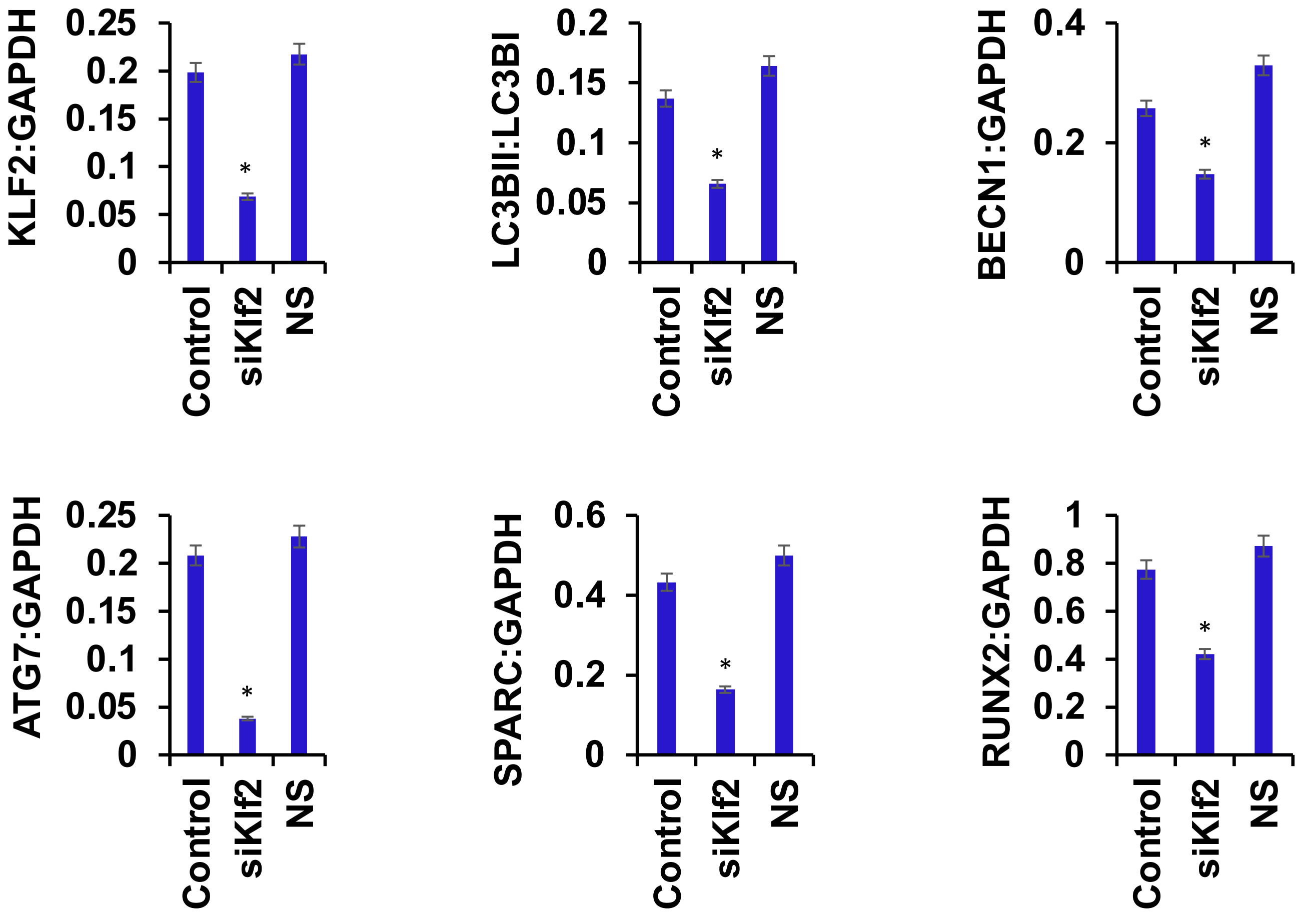
KLF2:GAPDH
0.25
0.2
0.15
0.1
0.05
0
*
Control
siKlf2
NS
LC3BII:LC3BI
0.2
0.15
0.1
0.05
0
*
Control
siKlf2
NS
BECN1:GAPDH
0.4
0.2
0
*
Control
siKlf2
NS
ATG7:GAPDH
0.25
0.2
0.15
0.1
0.05
0
*
Control
siKlf2
NS
SPARC:GAPDH
0.6
0.4
0.2
0
*
Control
siKlf2
NS
RUNX2:GAPDH
1
0.8
0.6
0.4
0.2
0
*
Control
siKlf2
NS

Figure S3

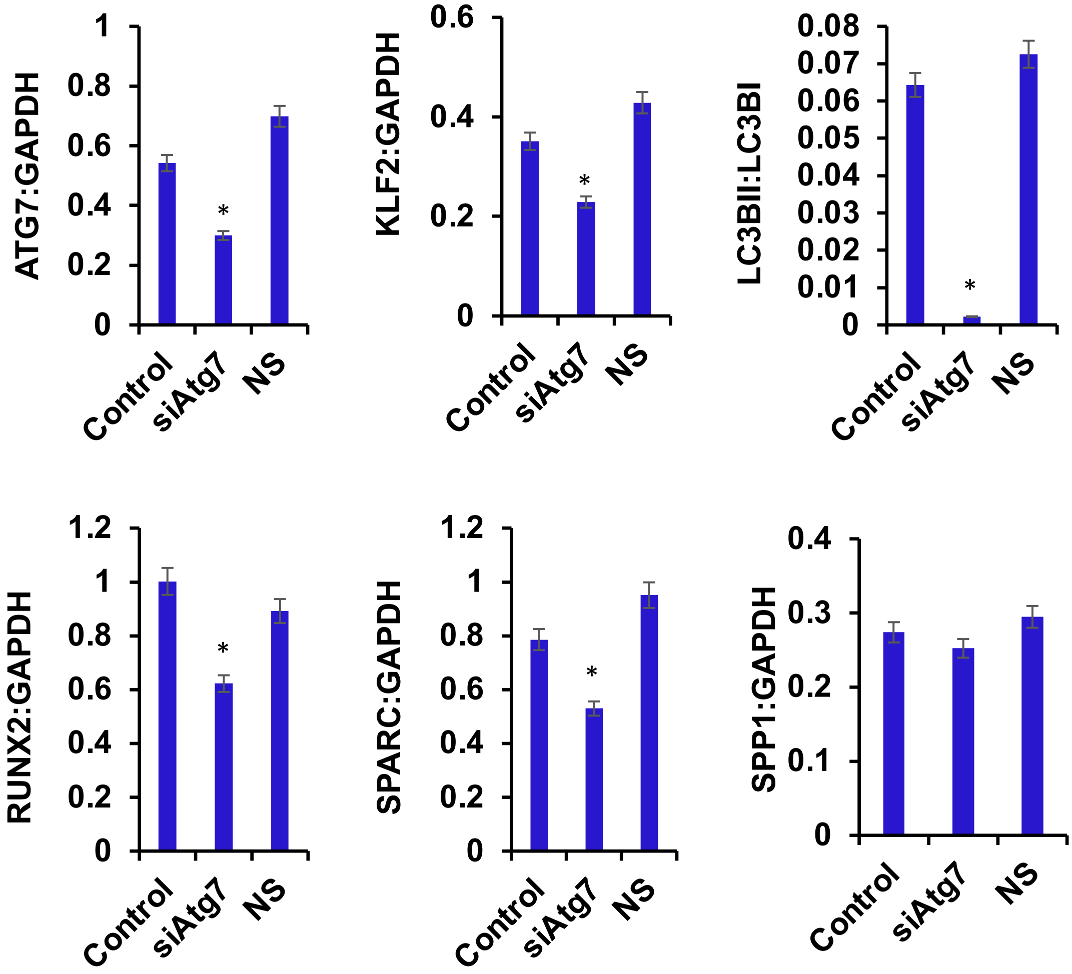
ATG7:GAPDH
1
0.8
0.6
0.4
0.2
0
*
Control
siAtg7
NS
KLF2:GAPDH
0.6
0.4
0.2
0
*
Control
siAtg7
NS
LC3BII:LC3BI
0.08
0.07
0.06
0.05
0.04
0.03
0.02
0.01
0
*
Control
siAtg7
NS
RUNX2:GAPDH
1.2
1
0.8
0.6
0.4
0.2
0
*
Control
siAtg7
NS
SPARC:GAPDH
1.2
1
0.8
0.6
0.4
0.2
0
*
Control
siAtg7
NS
SPP1:GAPDH
0.4
0.3
0.2
0.1
0
Control
siAtg7
NS

Figure S4

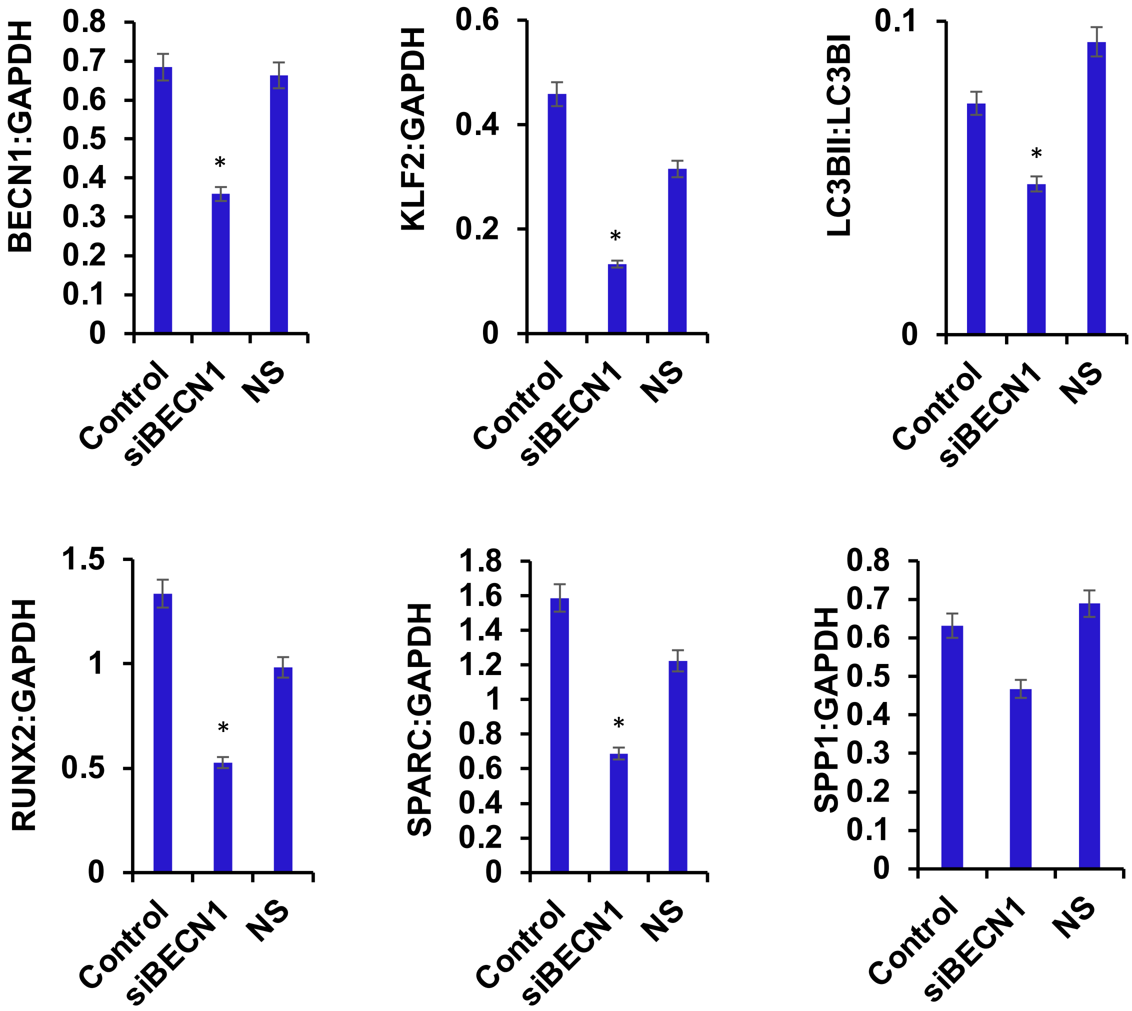
BECN1:GAPDH
0.8
0.7
0.6
0.5
0.4
0.3
0.2
0.1
0
*
Control
siBECN1
NS
KLF2:GAPDH
0.6
0.4
0.2
0
*
Control
siBECN1
NS
LC3BII:LC3BI
0.1
0
*
Control
siBECN1
NS
RUNX2:GAPDH
1.5
1
0.5
0
*
Control
siBECN1
NS
SPARC:GAPDH
1.8
1.6
1.4
1.2
1
0.8
0.6
0.4
0.2
0
*
Control
siBECN1
NS
SPP1:GAPDH
0.8
0.7
0.6
0.5
0.4
0.3
0.2
0.1
0
Control
siBECN1
NS

Figure S5

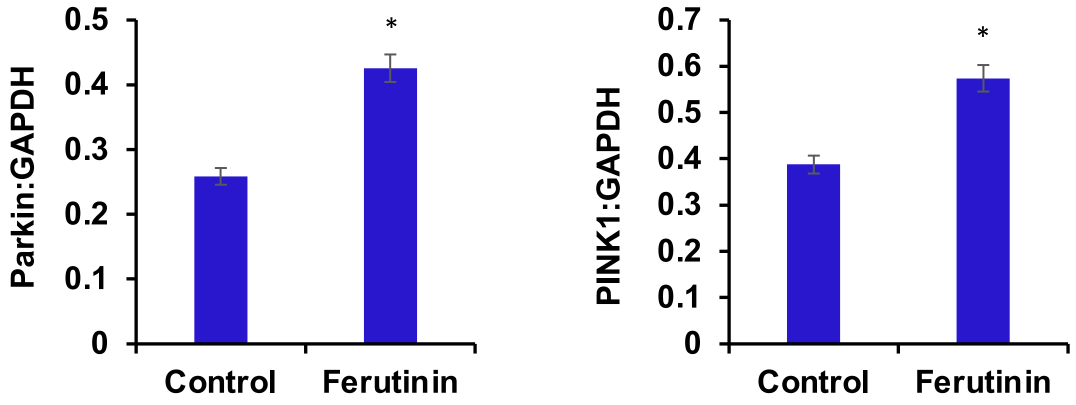
Parkin:GAPDH
0.5
0.4
0.3
0.2
0.1
0
*
Control
Ferutinin
PINK1:GAPDH
0.7
0.6
0.5
0.4
0.3
0.2
0.1
0
*
Control
Ferutinin

Figure S6

Highlighted regions were used for primer selection for Chromatin immunoprecipitation-PCR analysis

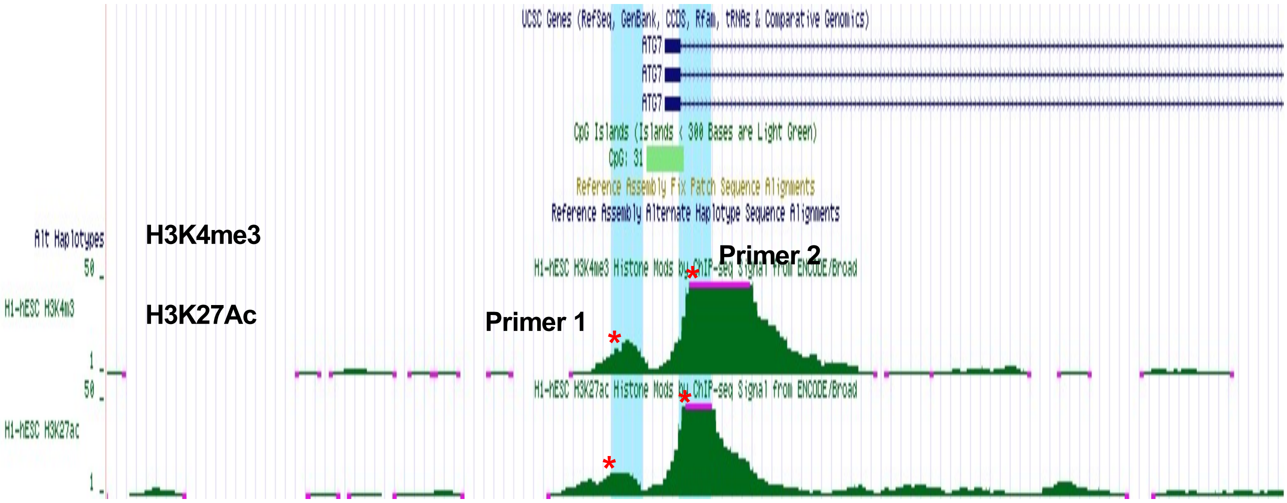

Figure S7

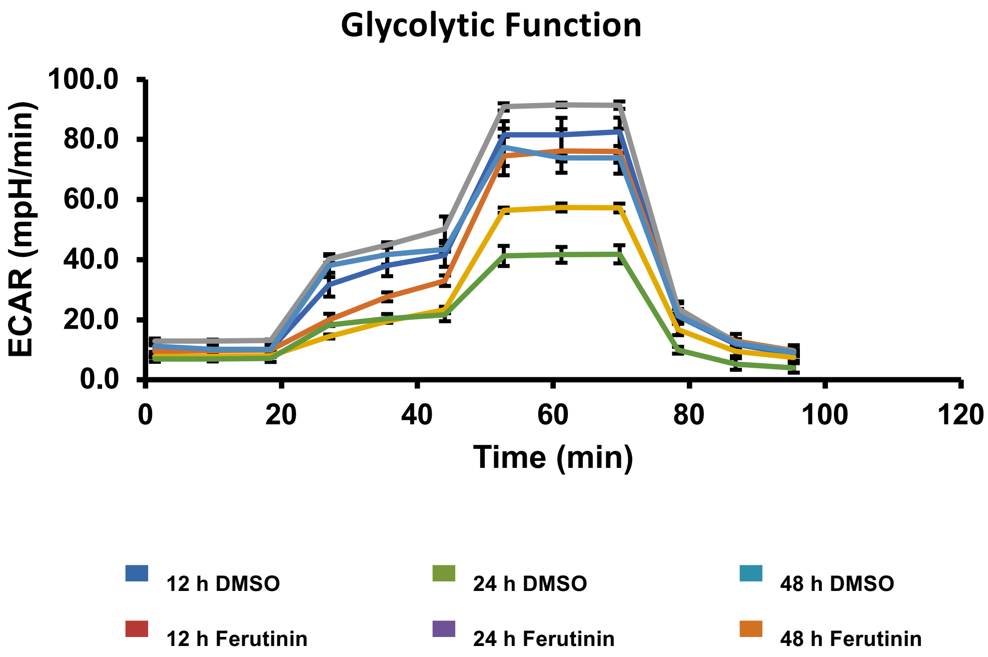
Glycolytic Function
ECAR (mpH/min)
100.0
80.0
60.0
40.0
20.0
0.0
0
20
40
60
80
100
120
Time (min)
12 h DMSO
24 h DMSO
48 h DMSO
12 h Ferutinin
24 h Ferutinin
48 h Ferutinin

Figure S8

Table S1. Primer sequences used in chromatin immunoprecipitation quantitative PCR assays

Primer	Name	Sequence	Genomic location from Human-GRCh37/hg19 assembly
1	**ATG7-1 Forward**	TTGAGCGGCGGTAAGTGAG	Chr3:11,313,635-11,313,654
2	**ATG7-1 Reverse**	CCTGAGACTTGCTGTGACCC	Chr3:11,313,758-11,313,778
3	**ATG7-1 Forward**	TTCTCATCTCCTGGCTCTCC	Chr3:11,314,180-11,314,200
4	**ATG7-1 Reverse**	TCTCCCTTACCAGATGTCGGA	Chr3:11,314,363- 11,314,384

Figure S9
